# Prediction of Human Phenotype Ontology terms by means of hierarchical ensemble methods

**DOI:** 10.1186/s12859-017-1854-y

**Published:** 2017-10-12

**Authors:** Marco Notaro, Max Schubach, Peter N. Robinson, Giorgio Valentini

**Affiliations:** 1 Anacleto Lab - Dipartimento di Informatica, Universitá degli Studi di Milano, Via Comelico 39, Milan, 20135 Italy; 2 Institute for Medical and Human Genetics, Charité - Universitätsmedizin Berlin, Augustenburger Platz 1, Berlin, 13353 Germany; 3 Max Planck Institute for Molecular Genetics, Ihnestraße 63-73, Berlin, 14195 Germany; 4 The Jackson Laboratory for Genomic Medicine, 10 Discovery Dr, Farmington, 06032 CT USA; 5 Institute for Systems Genomics, University of Connecticut, 10 Discovery Dr, Farmington, 06032 CT USA; 6 Berlin Institute of Health (BIH), Anna-Louisa-Karsch-Str. 2, Berlin, 10178 Germany

**Keywords:** Human Phenotype Ontology, Hierarchical multi-label classification, Hierarchical ensemble methods, Gene-Abnormal phenotype association, Human Phenotype Ontology term prediction, Phenotype gene prioritization

## Abstract

**Background:**

The prediction of human gene–abnormal phenotype associations is a fundamental step toward the discovery of novel genes associated with human disorders, especially when no genes are known to be associated with a specific disease. In this context the Human Phenotype Ontology (HPO) provides a standard categorization of the abnormalities associated with human diseases. While the problem of the prediction of gene–disease associations has been widely investigated, the related problem of gene–phenotypic feature (i.e., HPO term) associations has been largely overlooked, even if for most human genes no HPO term associations are known and despite the increasing application of the HPO to relevant medical problems. Moreover most of the methods proposed in literature are not able to capture the hierarchical relationships between HPO terms, thus resulting in inconsistent and relatively inaccurate predictions.

**Results:**

We present two hierarchical ensemble methods that we formally prove to provide biologically consistent predictions according to the hierarchical structure of the HPO. The modular structure of the proposed methods, that consists in a “flat” learning first step and a hierarchical combination of the predictions in the second step, allows the predictions of virtually any flat learning method to be enhanced. The experimental results show that hierarchical ensemble methods are able to predict novel associations between genes and abnormal phenotypes with results that are competitive with state-of-the-art algorithms and with a significant reduction of the computational complexity.

**Conclusions:**

Hierarchical ensembles are efficient computational methods that guarantee biologically meaningful predictions that obey the true path rule, and can be used as a tool to improve and make consistent the HPO terms predictions starting from virtually any flat learning method. The implementation of the proposed methods is available as an R package from the CRAN repository.

**Electronic supplementary material:**

The online version of this article (doi:10.1186/s12859-017-1854-y) contains supplementary material, which is available to authorized users.

## Background

In contrast to its general meaning that usually refers to the traits or characteristics of an organism, in medical contexts, the word “*phenotype*” is defined as a deviation from normal morphology, physiology, or behavior [1]. The analysis of phenotype is essential for understanding the pathophysiology of cellular networks and plays a key role in medical research and in the mapping of disease genes [2, 3]. The Human Phenotype Ontology (HPO) project [4] provides a standard categorization of the human abnormal phenotypes and of their semantic relationships. It is worth noting that each HPO term does not represent a disease, but rather denotes individual signs or symptoms or other clinical abnormalities that characterize a disease. The HPO is currently developed using the medical literature, and OMIM [5], Orphanet [6] and DECIPHER [7] databases, and contains approximately 11,000 terms and over 115,000 annotations to hereditary diseases. The HPO is structured as a direct acyclic graph (DAG), where more general terms are found on the top levels of hierarchy and the term specificity increases moving towards the lower levels of hierarchy, i.e. from root to leaves. As a consequence, differently from tree-structured taxonomies such as FunCat [8], each HPO term may have more than one parent. The HPO is governed by *true-path-rule* (also known as *annotation propagation rule*) [2]: if a gene is annotated with a given functional term, then it is annotated with all the “parent” terms, and with all its ancestors in a recursive way. On the contrary if a gene is not annotated to a term, it cannot be annotated to its offspring.

While the problem of the prediction of gene–disease associations has been widely investigated [9], the related problem of gene–HPO term prediction has been only considered in a few studies [10], despite the fact that no HPO term associations are known for most human genes, and the quickly growing application of the HPO to relevant medical problems [11, 12].

“Flat” classification methods have been applied to the prediction of gene-HPO term associations [13]. Unfortunately these methods can introduce major inconsistencies in the classification, because labels are independently predicted without taking into account the hierarchical relationships within the ontology [14]. For example, if we use the HPO to predict gene-phenotype relations, a flat learner can associate the HPO term “Hyperplasia of metatarsal bones” to a gene. But it might not associate the parent term “Abnormality of the metatarsal bones”, thus leading to an inconsistent prediction. In addition flat methods do not exploit a priori knowledge about the topology of the ontology, which may result in a reduction in the prediction accuracy.

To properly handle the hierarchical relationships between terms that characterize the HPO, we can apply two main classes of structured output methods, i.e. methods able to exploit in the learning process the hierarchical structure of terms [15]. The first category of methods exploits joint input and output kernelization techniques based on large margin methods for structured and interdependent output variables [16, 17]. The second general class of structured output methods is based on ensembles of learning machines able to exploit the hierarchical relationship between classes; theoretical studies [18], as well as applications in several domains [19] showed the effectiveness of this approach. Both these classes of methods have been applied to several bioinformatics problems, ranging from enzyme function prediction [17, 20] to the hierarchical prediction of Gene Ontology terms [21, 22]. In the context of HPO, Kahanda and coworkers [10] proposed a structured output method, based on a joint kernel constructed through the product of the input and the output kernel, and showed that the proposed approach outperforms existing methods.

To our knowledge no methods based on hierarchical ensembles have been proposed in the context of structured output prediction of HPO terms associated with human genes. Indeed most of the hierarchical ensemble methods proposed in literature are conceived for tree-structured taxonomies [23], and the few ones specific for DAGs have been mainly applied to the prediction of the gene and protein functions [22, 24]. Nevertheless, several of these methods, mainly proposed in the context of GO term classification, could be in principle applied to the prediction of HPO terms. For a review of these approaches we refer the reader to [15].

To fill this gap, we propose two distinct hierarchical ensemble methods, the *Hierarchical Top-Down (HTD-DAG)* and *True Path Rule (TPR-DAG)* for *Directed Acyclic Graphs (DAG)*, able to provide consistent predictions of HPO terms and to scale nicely both in terms of the complexity of the taxonomy and the cardinality of the examples. In this paper we show that the proposed approaches present several advantages with respect to state-of-the-art structured output methods, including their competitive accuracy together with low computational complexity, their modularity and capacity to enhance the predictions of both semi-supervised and supervised flat methods, and their ability to in principle provide any biologically consistent prediction of structured association between a gene and HPO terms.

A preliminary version of *HTD-DAG* and *TPR-DAG* were presented at *IWBBIO* and *MCS* workshops [25, 26], but these methods have not been previously published in any journal. Here for the first time we present a detailed explanation of the algorithms and provide formal proofs of the consistency of the predictions for both *HTD-DAG* and *TPR-DAG*. Moreover we introduce completely new variants, such as the Adaptive Threshold (AT) and the Descendant (D) algorithms (see below the “Methods” section for more details). We also discuss how the proposed methods can improve flat predictions, and we provide several examples of the biological consistency of the predictions obtained with both HTD and TPR-DAG algorithms. In the “Results and discussion” section we propose a completely new and enlarged experimental set-up involving a genome-wide experimental comparison of our proposed hierarchical ensembles with state-of-the-art methods for HPO term prediction. Finally we perform the prediction of new HPO annotations based on earlier annotations and we also propose novel HPO annotations for several genes, showing how such predictions are confirmed by the literature or by up-to-date HPO annotations not used during the training of the algorithm.

## Methods

We present two algorithms, *Hierarchical Top-Down (HTD-DAG)* and *True Path Rule (TPR-DAG)* for *Directed Acyclic Graphs (DAG)*, specifically designed to exploit the DAG structure of the relationships between HPO terms to predict associations between genes and sets of HPO terms. Both hierarchical ensemble methods adopt a two-step learning strategy:

1. *Flat learning of the terms of the ontology*: each base classifier learns a specific and individual class (HPO term) resulting in a set of dichotomic classification problems which are independent of each other.
2. *Hierarchical combination of the predictions*: aggregation of the classifications performed by the base learners trained in the previous step. The resulting consensus predictions of the ensemble take the hierarchical relationships of the ontology into account.

This ensemble approach is highly modular: in principle any learning algorithm can be used to train the classifiers in the first step, and in the second step the hierarchical relationships between the HPO terms are exploited to achieve the final ensemble predictions of the set of HPO terms associated with a specific gene.

The main limitation of the flat learning of the HPO terms is that each term is separately learned without taking into account the relationships between classes. Other methods, such as the ensemble approach proposed in [27] can overcome this limitation by applying global models for the multi-label classification, thus explicitly considering class interactions during the learning process. On the other hand hierarchical ensemble methods are able to correct flat predictions, by splitting in two separate steps the process of learning HPO terms and the process of evaluating the hierarchical relationships between classes.

### Basic notation and definitions

The directed acyclic graph *G*=<*V*,*E*> represents the HPO taxonomy, its vertices *V*={1,2,…,|*V*|} the terms of the ontology and the directed edges (*i*,*j*)∈*E* the hierarchical relationships between the parent terms *i* and their children *j*. We define *c* *h* *i* *l* *d*(*i*) as the children of a term *i*, *p* *a* *r*(*i*) as the set of its parents, *a* *n* *c*(*i*) as its ancestors and *d* *e* *s* *c*(*i*) as the set of its descendants.

A “flat multi-label scoring” predictor $f: X \rightarrow \mathbb {Y}$ provides a score $f(x) = \hat {\boldsymbol {y}}, \hat {\boldsymbol {y}} \in \mathbb {Y}=[0,1]^{|V|}$ for a given example *x*∈*X*, where *X* is a suitable input space for the predictor *f*. In other words a flat predictor provides a score $\hat {y}_{i} \in [0,1]$ that represents the likelihood that a given gene belongs to a given node/HPO term *i*∈*V* of the DAG *G*, and $\hat {\boldsymbol {y}} = < \hat {y}_{1}, \hat {y}_{2}, \ldots, \hat {y}_{|V|}>$. We say that the multi-label scoring ***y*** is consistent if it obeys the *true path rule*:

1 $$ \boldsymbol{y} \; \text{is \; consistent} \iff \forall i \in V, j \in par(i) \Rightarrow y_{j} \geq y_{i}   $$

It is straightforward to show that (1) holds even with flat classifiers that do not provide a score but a label $\hat {y}_{i} \in \{0,1\}$ indicating that a given gene belongs ($\hat {y}_{i} = 1$) or does not ($\hat {y}_{i} = 0$) to a given HPO term *i*.

It is very unlikely that the true path rule could be satisfied by a flat multi-label scoring predictor, but with an additional topology-aware score/label modification step we can easily satisfy the constraints imposed by the true path rule. More precisely, we can provide a prediction function $g\left (f(x)\right): \mathbb {Y} \rightarrow \mathbb {Y}$ such that the *true path rule* (1) holds for all the predictions $g\left (f(x)\right) = \bar {\boldsymbol {y}}$: $\forall i \in V, j \in par(i) \Rightarrow \bar {y}_{j} \geq \bar {y}_{i}$.

### Flat learning of the terms of the ontology

The algorithm first utilizes a flat ensemble learning strategy by which each term *i*∈*V* of the HPO is independently learned through a term specific predictor *f*_*i*_:*X*→[0,1]. Accordingly, the output of the flat classifier $f:X \rightarrow \mathbb {Y}$ on the instance *x*∈*X* is $f(x) = \hat {\boldsymbol {y}}$:

$${}f(x) = <f_{1}(x),\ f_{2}(x),\ \ldots,\ f_{|V|}(x)>= <\hat{y}_1,\hat{y}_2,\ \ldots,\ \hat{y}_{|V|}>$$

To this end any supervised or semi-supervised base predictor can be used, including also flat binary classifiers. Indeed both learners able to provide a probability or a score related to the likelihood that a gene is annotated with a HPO term (that is scores $\hat {y}_{i} \in [0,1]$), and base binary classifiers that can directly provide a label (but not a score) about the association gene-HPO term (that is a label $\hat {y}_{i} \in \{0,1\}$) can be used to generate the flat predictions. Note that the training of per-class predictors *f*_1_, *f*_2_, …,*f*_|*V*|_ can be performed in parallel, and it is easy to achieve a linear speed-up in the number of the available processors by adopting simple parallel computational techniques.

For the HPO predictions we used semi-supervised (*RANKS*, [28]) and supervised (Support Vector Machines – SVM [29]) machine learning methods to implement the base learners of the proposed hierarchical ensemble methods.

The semi-supervised approach RAnking of Nodes with Kernalized Score functions (*RANKS*) is a network-based method, that adopts a local as well as a global prediction strategy. By using local learning, *RANKS* measures the similarity between a gene and its neighbors using different score functions. Global learning is accomplished through graph kernels that exploit the overall topology of the network to predict node labels. In principle any valid kernel function can be applied here. In this work we apply the average score function and a 1, 2 and 3-step random walk kernels (that are respectively able to explore direct neighbors and genes at 2 or three steps away in the network). *RANKS* was previously successfully applied in the prioritization of disease genes [30], the prediction of gene function [31] as well as for drug repositioning problems [32].

It is worth noting that *RANKS* returns a score and not a probability, that represents the likelihood that a gene belongs to a given class, but the “magnitude” of the scores may vary across different classes [31]. To make the scores comparable across classes, we considered two distinct normalization procedures:

1. Normalization in the sense of the maximum: the score of each class is normalized by dividing the score values for the maximum score of that class;
2. Quantile normalization: a method originally designed for the normalization of probe intensity levels for high density oligonucleotide microarray data across multiple experiments [34]. In our case we applied quantile normalization to make the scores comparable across different HPO terms.

SVMs were trained for each term using the R interface of the machine learning library *LiblineaR* [35] with default parameter settings. Because of the high running time of SVMs we implemented a *multicore* version of *LiblineaR* using *doParallel* and *foreach* R packages. The parallel implementation of SVMs is available upon request from the authors.

### Hierarchical Top-Down (*HTD-DAG*) ensembles

**Fig. 1:**
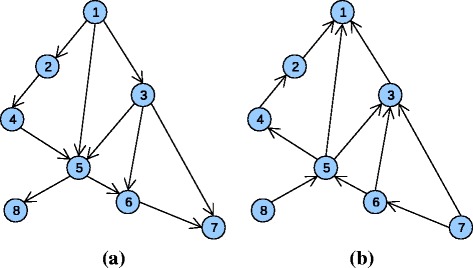
Flow of information in hierarchical ensembles. **a** Top-down flow, **b** Bottom-up flow. See text for more explanations

The main idea behind the Hierarchical top-down algorithm *(HTD-DAG)* consists in modifying the predictions of each base learner from “top to bottom”, i.e. from the least to the most specific terms by exploiting at each step the predictions provided by the less specific predictors, e.g. predictors associated to parent HPO terms. This is performed in a recursive way by transmitting the predictions from each node to the their children, and from the children to the children of the children through a propagation of the information towards the descendants of each node of the ontology. For instance in Fig. 1 a the information can flow along the path traversing nodes 1,5,6,7 or 1,3,7, and a prediction for e.g. the node 5 depends on the predictions performed by the base learners for the parent nodes 4,1 and 3. This operating mode of the ensemble is performed in an ordered way from the top to the bottom nodes (Fig. 1a).

More precisely, the *HTD-DAG* algorithm modifies through a unique run across the nodes of the graph the flat scores according to the hierarchy of a DAG. The flat predictions $f(x) = \hat {\boldsymbol {y}}$ are hierarchically corrected to $\bar {\boldsymbol {y}}$ by top-down per-level traversing the nodes of the DAG, and by applying recursively this rule:

2 $$ \bar{y}_{i} := \left\{ \begin{array}{lll} \hat{y}_{i} & \text{if} \quad i \in root(G) \\ \min_{j \in par(i)} \bar{y}_{j} & \text{if} \quad \min_{j \in par(i)} \bar{y}_{j} < \hat{y}_{i} \\ \hat{y}_{i} & \text{otherwise} \end{array} \right.   $$

The maximum path length from the root is used to define the node levels. More precisely, given that *p*(*r*,*i*) represents a path from the root node *r* and a node *i*∈*V*, *l*(*p*(*r*,*i*)) the length of *p*(*r*,*i*), $\mathcal L = \{0, 1, \ldots, \xi \}$ the observed levels, and *ξ* the maximum node level, then we can define a level function $\psi : V \longrightarrow \mathcal L$:

3 $$ \psi(i) = \max_{p(r,i)}\ l \left(p(r,i)\right)   $$

The above level function *ψ* assigns each node *i*∈*V* to its level *ψ*(*i*). For instance, from this definition root nodes are {*i*|*ψ*(*i*)=0}, while nodes lying at a maximum distance *ξ* from the root are {*i*|*ψ*(*i*)=*ξ*}.

The consistency of the predictions is guaranteed if and only if the levels are defined according to the maximum path length from the root (Correctness and consistency of the predictions section provides a formal proof of this fact).

**Fig. 2:**
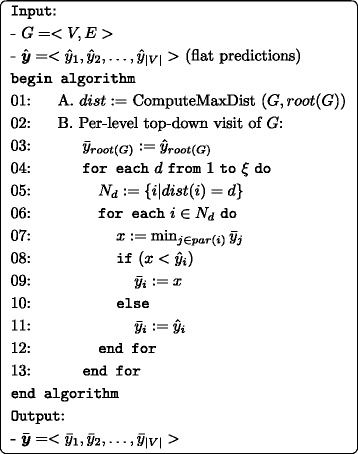
The Hierarchical Top-Down algorithm for DAGs (HTD-DAG)

Figure 2 shows the pseudo code of the second step of *HTD-DAG* algorithm, by which the flat predictions $\hat {\boldsymbol {y}}$ computed in the first step are combined and updated according to top-down per-level traversal of the DAG.

The block A of the algorithm (row 1) computes the maximum distance of each node from the root; to this end the classical Bellman-Ford algorithm or the methods based on the Topological Sorting algorithm can be applied [36].

A top-down per-level traversal of the graph is implemented in the block B: for each level of the graph, starting from the nodes just below the root, the flat predictions $\hat {y}_{i}$ are top-down corrected, according to Eq. 2, and the consensus ensemble predictions $\bar {y}_{i}$ are obtained. More precisely, the nested loops starting respectively at line 04 and 06 ensure that nodes are processed by level in an increasing order. Lines 07–11 perform the hierarchical correction of the flat predictions $\hat {y}_{i}$, *i*∈{1,…,|*V*|}. The algorithm ends when nodes at distance *ξ* from the root are processed (last iteration of the external loop within lines 04–13) and it finally provides the hierarchically corrected predictions $\bar {\boldsymbol {y}}$.

The complexity of block *A* is $\mathcal {O}(|V| + |E|)$ (if the Topological Sort algorithm is used to implement *ComputeMaxDist*), while it is easy to see that the complexity of block *B* (rows 3−13) is $\mathcal {O}(|V|+|E|)$. Hence the overall complexity of the top-down step of *HTD-DAG* is $\mathcal {O}(|V|+|E|))$, that is linear in the number of nodes of the HPO, considering that its underlying DAG is sparse.

### Hierarchical true path rule (*TPR-DAG*) ensembles

By considering the opposite flow of information “from bottom to top”, we can construct the prediction of the ensemble by recursively propagating the predictions provided by the the most specific nodes toward their parents and ancestors.

**Fig. 3:**
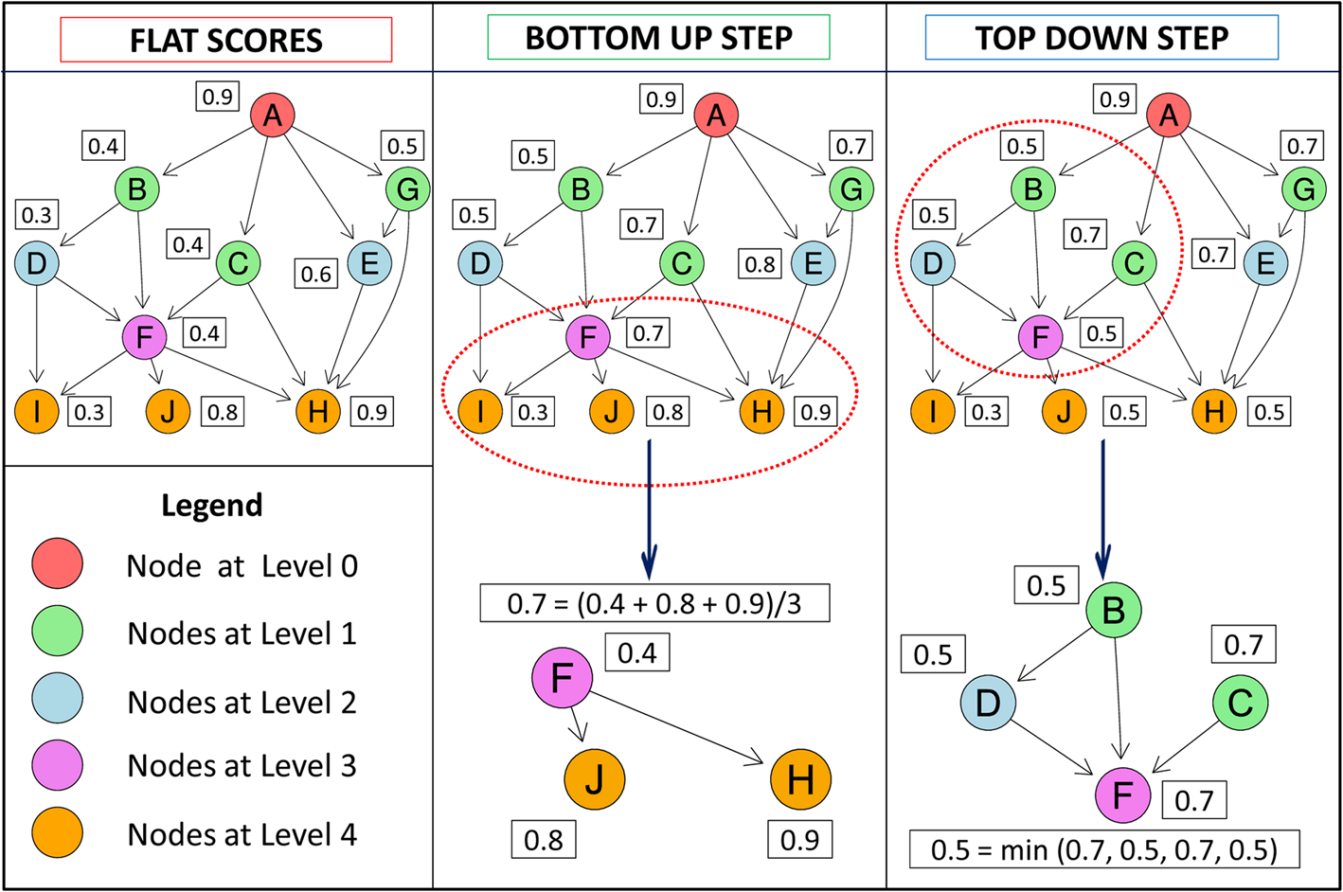
A toy example of the operating mode of the *TPR-DAG* method. Left: The nodes represent different HPO terms and the numeric values the flat scores associated to each node of the graph. The different colors represent the levels, i.e. the maximum distance of the node from the root node *A*. Center: The bottom-up step introduces a correction of the flat scores by taking into account the scores of the children of each node. This procedure is methodically repeated from the bottom to the top nodes of the DAG; as an example, the bottom part shows that the correction for the *F* node is performed by averaging the flat score of the *F* parent node with those of the “positive” children, i.e. that children nodes having a value larger than that of the *F* parent node. Right: The Top-down step introduces a further correction by taking into account the scores of the parent nodes, by methodically parsing this time the DAG from the root node *A* down to descendant nodes; as an example, the bottom part of the figure shows that the score of the *F* node is set to the minimum of the bottom-up scores of *F* and that of its parents *A*, *B* and *C*

For instance in Fig. 1b a possible flow of information could be along the path 8,5,4,2,1 or 7,6,5,1, and the prediction of the ensemble for e.g. node 3 depends on children nodes 5,6 and 7. The proposed True Path Rule for DAG (*TPR-DAG*) adopts this bottom-up flow of information, to take into account the predictions of the most specific HPO terms, but also the opposite flow from top to bottom to consider the predictions of the least specific terms. Figure 3 provides a pictorial toy example of the operating mode of the *TPR-DAG* algorithm.

This algorithm is related to the *TPR* algorithm for tree-structured taxonomies [37], but despite the similarity of their names, the *TPR* for trees cannot be applied to the HPO, since it does not work on DAG-structured taxonomies and provides inconsistent predictions when applied to the HPO. In contrast to the per-level tree traversal proposed in [37], the DAG per-level traversal has two distinct and strictly separated steps: (1) the DAG is inspected bottom-up per-level, followed by (2) a top-down visit. This separation is necessary to assure the true path rule consistency of the predictions in DAG-structured taxonomies (see “Correctness and consistency of the predictions” section for details). The other main difference consists in the way the levels are computed: in this new DAG version the levels are constructed according to the maximum distance from the root, since this guarantees that in the top-down step all the ancestor nodes have been processed before their descendants, thus assuring the true path rule consistency of the predictions (see “Correctness and consistency of the predictions” section for a formal proof of this fact). Moreover in this paper we also propose novel algorithms for the bottom-up propagation of the predictions from the most to the least specific terms of the HPO.

*TPR-DAG* provides “consensus” ensemble predictions by integrating the flat predictions $\hat {y}_{i}$ through a per-level visit of the DAG:

4 $$ \bar{y}_{i} := \frac{1}{1 + |\phi_{i}|} \left(\hat{y}_{i} + \sum_{j \in \phi_{i}} \bar{y}_{j}\right)   $$

where *ϕ*_*i*_ are the “positive” children of *i*.

Note that only positive predictions of the children obey the true path rule. Indeed, according to this rule, we may have a gene annotated to a term *t*, but not annotated to a terms *s*∈*c* *h* *i* *l* *d*(*t*). Hence if we have a negative prediction for the terms *s* it is not meaningful to use this prediction to predict the term *t*. It is worth noting that we can combine children predictions using aggregation strategies other than the average. For instance using the maximum, we could likely improve the sensitivity, but with a likely decrement of the precision. Different strategies to select the “positive” children *ϕ*_*i*_ can be applied, according to the usage of a specific threshold to separate positive from negative examples:

1. *Constant Threshold (T) strategy.* For each node the same threshold $\bar {t}$ is a priori selected: $t_{j} = \bar {t}, \quad \forall j \in V$. In this case ∀*i*∈*V* we have:
  5 $$ \phi_{i} := \{ j \in child(i) | \bar{y}_{j} > \bar{t} \}   $$
  For instance if the predictions represent probabilities it could be meaningful to a priori select $\bar {t} = 0.5$.
2. *Adaptive Threshold (AT) strategy.* The threshold is selected to maximize some performance metric $\mathcal {M}$ estimated on the training data, e.g. the F-score or the AUROC. In other words the threshold is selected to maximize some measure of accuracy of the predictions $\mathcal {M}(j,t)$ on the training data for the class *j* with respect to the threshold *t*. The corresponding set of positives ∀*i*∈*V* is:
  6 $$ \phi_{i} := \left\{ j \in child(i) | \bar{y}_{j} > t_{j}^{*}, t_{j}^{*} = \arg \max_{t} \mathcal{M}(j,t) \right\}   $$
  For instance internal cross-validation can be used to select $t^{*}_{j}$ from a set of *t*∈(0,1).
3. *Threshold Free (TF) strategy.* This strategy does not require an a priori or experimentally selected threshold. We select as positive those children that increment the score of their parent node *i*:
  7 $$ \phi_{i} := \{ j \in child(i) | \bar{y}_{j} > \hat{y}_{i} \}   $$

Accordingly, we can derive three different algorithmic variants of the basic *TPR*:

- *TPR-T*: *TPR* with constant threshold, corresponding to strategy 1);
- *TPR-AT*: *TPR* with adaptive thresholds, corresponding to strategy 2);
- *TPR-TF*: *TPR* threshold-free, corresponding to strategy 3).

**Fig. 4:**
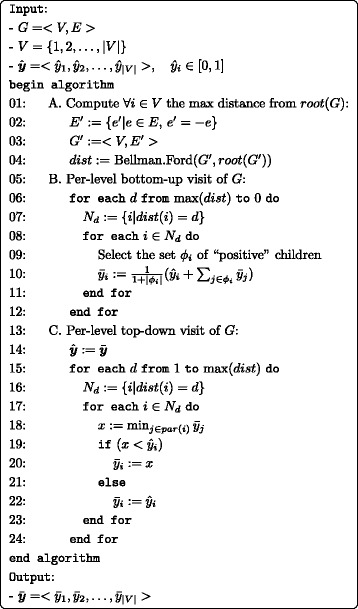
Hierarchical True Path Rule algorithm for DAGs (TPR-DAG)

All three *TPR* algorithms move positive predictions recursively towards the ancestors so that the predictions are propagated bottom-up. The pseudo-code of the TPR-DAG algorithm is shown in Fig. 4 and it is structured into three parts. At first in part A, the maximum distance for every node *V*_*i*_ to the root is computed via the Bellman-Ford algorithm. Second, block B updates the predictions $\hat {y}_{i}$ using Eq. 4 together with one of the three positive selection strategies by a bottom-up visit. After this step the true path rule have to be fulfilled, since the bottom up step assures the propagation of positive predictions, but not their consistency. To this end, in the final block C, the hierarchical top-down step is performed, like in the *HTD-DAG* algorithm.

The complexity of the *TPR-DAG* algorithm is quadratic in the number of nodes for the block *A*, but can be $\mathcal {O}(|V|+|E|)$ if the Topological Sort algorithm is used instead. It is easy to see that the complexity is $\mathcal {O}(|V|)$ for both the *B* and *C* blocks when graphs are sparse. Hence, considering the sparseness of the HPO, the algorithm is linear with respect to the number of the terms of the HPO.

To modulate the contribution to the ensemble prediction of the parent node and its children, a *TPR-DAG* variant similar to the weighted True Path Rule algorithms for tree-structured taxonomies [23] can be designed for DAGs. The *TPR-W*, i.e. *TPR*-Weighted, can be obtained by weighting the predictions according to this rule:

8 $$ \bar{y}_{i} := w \hat{y}_{i} + \frac{(1 - w)}{|\phi_{i}|} \sum_{j \in \phi_{i}} \bar{y}_{j}   $$

In this approach a weight *w*∈[0,1] is added to balance between the contribution of the node *i* and that of its “positive” children. If *w*=1 no weight is attributed to the children and the *TPR-DAG* reduces to the *HTD-DAG* algorithm, since in this way only the prediction for node *i* is used in the bottom-up step of the algorithm. If *w*=0 only the predictors associated to the children nodes “vote” to predict node *i*. In the intermediate cases we attribute more importance to the predictor for the node *i* or to its children depending on the values of *w*. A different way to implement a weighting strategy could be also pursued not only considering balancing between the predictions on node *i* and nodes *j*∈*c* *h* *i* *l* *d*(*i*), but including also weighting with respect to the estimated accuracy of each base learner, estimated e.g. by internal cross-validation.

As shown in [37] for the tree version of the *TPR* algorithm, the contribution of the descendants of a given node decays exponentially with their distance from the node itself, and it is easy to see that this is true also for the *TPR-DAG* algorithm. To enhance the contribution of the most specific nodes to the overall decision of the ensemble, a linear decaying or a constant contribution of the “positive” descendants could be considered instead:

9 $$ \bar{y}_{i} := \frac{1}{1 + |\Delta_{i}|} \left(\hat{y}_{i} + \sum_{j \in \Delta_{i}} \bar{y}_{j}\right)   $$

where

10 $$ \Delta_{i} = \left\{ j \in desc(i) | \bar{y}_{j} > t_{j} \right\}   $$

In this way all the “positive” descendants of node *i* provide the same contribution to the ensemble prediction $\bar {y}_{i}$. We named this *TPR* variant as *TPR-D*.

### Correctness and consistency of the predictions

Hierarchical ensemble methods can improve flat predictions by reducing the number of both false positives (*FP*) and false negatives (*FN*). For instance, the Additional file 1 (Figure S1a) shows that hierarchical ensembles can correct *FN* flat predictions to *TP* for the gene *RGS9* (regulator of G-protein signaling 9) that encodes a member of the RGS family of GTPase whose mutations cause bradyopsia [38], while the Additional file 1: Figure S1b of the same additional file shows the ability of hierarchical ensembles of correcting *FP* to *TN* for the gene *ENAM* (enamelin) that encodes the largest protein in the enamel matrix whose deficiency is associated with amelogenesis imperfecta type 1C [39]. More precisely, in the Additional file 1: Figure S1a the hierarchical ensemble *TPR-W* “recovered” four *TP* (red rectangles), while in Additional file 1: Figure S1b *TPR-W* corrected six *FP* to *TN*. It is worth nothing that the hierarchical ensemble methods can improve the correctness, but they cannot of course guarantee the correctness of all the predictions: when, e.g. the flat predicted scores are too bad, hierarchical ensembles may fail in improving the recovery of *FP* or *FN*. For instance, in Additional file 1: Figure S1a *TPR-W* failed in removing three *FN* (orange rectangles), and in Additional file 1: Figure S1b was not able to remove three *FP*.

**Fig. 5:**
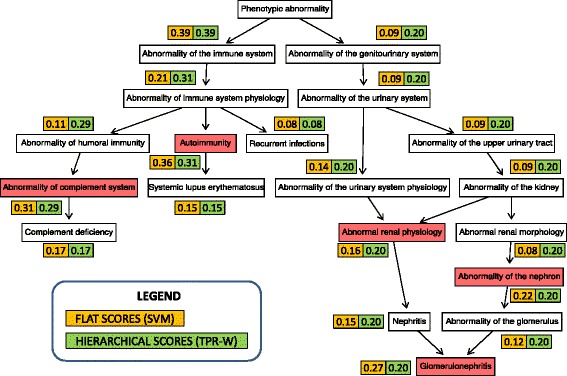
Flat and hierarchical (*TPR-W*) HPO predictions for the gene *C1QC*. The numbers close to each predicted HPO term represent flat (yellow rectangles) and hierarchically corrected (green) scores. The *TPR-W* predictions obey the true-path rule (the scores of the parent nodes are always larger or equal than that of their children nodes), while flat predictions are inconsistent for 5 HPO terms highlighted in light-red: Autoimmunity, Abnormality of complement system, Abnormal renal physiology, Abnormality of the nephron and Glomerulonephritis

Hierarchical ensemble methods can guarantee consistent predictions, that is predictions that obey the true path rule. To this end, the per-level visit of the hierarchical taxonomy should be realized by using the maximum and not the minimum distance from the root (see the Additional file 2 for an intuitive example showing this fact). An example of the capability of obtaining hierarchically corrected consistent predictions from inconsistent flat predictions is shown in Fig. 5 for the gene *C1QC* (complement C1q C chain), that encodes a 18 polypeptide chains protein whose deficiency is associated with lupus erythematosus and glomerulonephritis [40].

The consistency of the predictions of both the *HTD-DAG* and *TPR-DAG* is proved through the following theorems (proofs are available in the Additional file 3):

#### **Theorem 1**

Given a DAG *G*=<*V*,*E*>, a level function *ψ* that assigns to each node its maximum path length from the root and the set of *HTD-DAG* flat predictions $\hat {\boldsymbol {y}} = < \hat {y}_{1}, \hat {y}_{2}, \ldots, \hat {y}_{|V|}>$, the top-down hierarchical correction of the *HTD-DAG* algorithm assures that the set of ensemble predictions $\bar {\boldsymbol {y}} =< \bar {y}_{1}, \bar {y}_{2}, \ldots, \bar {y}_{|V|}>$ satisfies the following property: $\forall i \in V, \; j \in par(i) \Rightarrow \bar {y}_{j} \geq \bar {y}_{i}$

An immediate consequence of this theorem is that *HTD-DAG* assures consistent predictions not only for the parents, but also for all the ancestors of any node of a hierarchical DAG-structured taxonomy:

#### **Corollary 1**

Given a DAG *G*=<*V*,*E*>, the level function *ψ* and the set of flat predictions $\hat {\boldsymbol {y}} = < \hat {y}_{1}, \hat {y}_{2}, \ldots, \hat {y}_{|V|}>$, the *HTD-DAG* algorithm assures that for the set of ensemble predictions $\bar {\boldsymbol {y}} = < \bar {y}_{1}, \bar {y}_{2}, \ldots, \bar {y}_{|V|}>$ the following property holds: $\forall i \in V, \; j \in anc(i) \Rightarrow \bar {y}_{j} \geq \bar {y}_{i}$.

Independently of the choice of the positive children (“Hierarchical true path rule (*TPR-DAG*) ensembles” subsection), the following consistency theorem holds for *TPR-DAG*:

#### **Theorem 2**

Given a DAG *G*=<*V*,*E*>, a set of flat predictions $\hat {\boldsymbol {y}} = < \hat {y}_{1}, \hat {y}_{2}, \ldots, \hat {y}_{|V|}>$ for each class associated to each node *i*∈{1,…,|*V*|}, the *TPR-DAG* algorithm assures that for the set of ensemble predictions $\bar {\boldsymbol {y}} = < \bar {y}_{1}, \bar {y}_{2}, \ldots, \bar {y}_{|V|}>$ the following property holds: $\forall i \in V, \; j \in anc(i) \Rightarrow \bar {y}_{j} \geq \bar {y}_{i}$.

Finally a good property of *TPR-DAG* is that its sensitivity is always equal or better than that of the *HTD-DAG*:

#### **Theorem 3**

The *TPR-DAG* ensemble algorithm with “positive” children selected according to (7) achieves always a sensitivity equal or higher than the *HTD-DAG* ensemble algorithm.

Unfortunately there is no guarantee that the precision of *TPR-DAG* is always larger or equal than that of the *HTD-DAG* algorithm.

All the proofs and details about the above theorems are available in the Additional file 3.

## Results and discussion

We performed two different sets of experiments to compare our proposed hierarchical ensemble approach with state-of-the-art methods for the prediction of abnormal human phenotypes structured according to the HPO. In the first set of experiments (“Prediction of Human Phenotype Ontology terms” subsection) we compared *HTD* and *TPR* ensembles with state-of-the-art methods using the same data and experimental set-up adopted by [10]. In the second set of experiments (“HPO Prediction of newly annotated genesHPO Predic-” subsection) we evaluated the ability of our proposed hierarchical ensemble methods to predict newly annotated genes of the April 2016 HPO release, by using annotations of a previous release (January 2014). Finally we provided a list of currently unannotated genes that could be possible candidate genes for novel annotations, on the basis of the predictions performed by our proposed hierarchical ensemble methods.

### Prediction of Human Phenotype Ontology terms

We compared the hierarchical ensemble methods for DAGs (*HTD*, “Hierarchical Top-Down (*HTD-DAG*) ensembles” subsection) and *TPR* and its variants, (“Hierarchical true path rule (*TPR-DAG*) ensembles” subsection) against several state-of-the-art and baseline methods:

- PHENOstruct, a state-of-the art joint-kernel structured support vector machine approach [10]. This method uses the product of the input and output space kernel to construct the joint kernel. The rationale behind this approach is that two input/output pairs are considered similar if they are similar in both their input feature space and their output label space.
- Clus-HMC-Ens, a state-of-the art Hierarchical Multilabel classification (HMC) based on decision tree ensembles [27]. Differently from the proposed *HTD-DAG* and *TPR-DAG* methods, where each base learner solves a separate binary classification problem, each decision tree in the Clus-HMC-Ens ensemble is a “global” model built to predict all classes at the same time, thus allowing to explicitly take into account the relationships between the classes just at the level of each base learner.
- *SSVM* →*disease* →*HPO* *method*, an indirect two-step method that first predicts gene-disease associations and then maps them to HPO terms using the associations available on the HPO website [10].
- *PhenoPPIOrth*, a computational tool that can predict a set of OMIM diseases for given human genes using protein-protein interaction and orthologous proteins data and then maps the predicted OMIM terms to HPO terms by direct mapping [13].
- Probabilistic support vector machines (SVMs) [41]. This is a variant of the classical SVM algorithm, by which the output of the SVM is fitted to a sigmoid in order to provide an estimation of the probability that a given example belongs to the class to be predicted.
- RANKS, a semi-supervised method base on kernelized score functions [28], resulted one of the top-ranked methods in the recent CAFA2 challenge for HPO term prediction [42]. This is a semi-supervised node-label ranking algorithm that applies graph kernels to the gene network to exploit its global topological characteristics, and then simple local learning functions on the resulting kernelized graph to provide a ranking of the nodes (genes).

We used both a semi-supervised (*RANKS* [28]) and a supervised (Support Vector Machines – SVM) machine learning method to implement the base learners of the proposed hierarchical ensemble methods (see “Flat learning of the terms of the ontology” subsection for more details).

#### Data and experimental set-up

We used the same data and the same experimental set-up applied in [10] for a fair comparison with previously proposed methods [10, 13, 27]. Indeed, to our knowledge, [10] presented one of the largest comparative evaluation of different methods for the prediction of HPO terms at genome-wide level. Moreover *PHENOStruct*, described in the same paper, was one one of the top-ranked method in the recent CAFA2 challenge [42]. The generalization performance of the methods was evaluated through a classical 5-fold cross-validation procedure, according to [10].

It is worth noting in the training phase we selected the negative examples (genes) according to a “basic” selection strategy [23], i.e. for positives we used all the instances annotated with that HPO term, while for negatives we simply used all the not annotated instances. In principle other more refined strategies to select negatives may lead to improved performances [43, 44].

##### Data sources

We used the same version of the the STRING (v. 9.1, [45]) and BioGRID (v. 3.2.106, [46]) databases used in [10]. More precisely we downloaded physical and genetic experimental interactions relative to 4970 proteins from BioGRID 3.2.106, and the integrated protein-protein interaction and functional association data for 18172 human proteins from STRING 9.1. From the same STRING website we also downloaded the protein aliases file to map proteins to genes, using as identifiers respectively the Locus STRING ID and the ENTREZ Gene ID. Moreover, starting from the Gene Ontology annotations for the three main sub-ontologies (Biological Process, Molecular Function and Cellular Component [47]) and from OMIM annotations [48], both represented as binary feature vectors, we constructed 4 more networks by using the classical Jaccard index to represent the edge weight (functional similarity) between the nodes (genes) of the resulting network. In our context the Jaccard index of two genes measures the ratio between the cardinality of their common annotations and the cardinality of the union of their annotations. The rationale behind the usage of this index is that two genes are similar if they share most of their annotations. All these annotations were obtained by parsing the raw text annotation files made available by Uniprot knowledge-base considering only its SWISSprot component (release May 2013). Finally the resulting *n*=6 networks were integrated by averaging the edge weights $w^{d}_{ij}$ between the genes *i* and *j* of each network *d*∈{1,*n*} after normalizing their weights in the same range of values $w^{d}_{ij} \in [0,1]$ (*Unweighted Average* (UA) network integration, [30]):

11 $$ \bar{w}_{ij} = \frac{1}{n} \sum_{d = 1}^{n} w_{ij}^{d}   $$

The weighted adjacency matrices representing the obtained networks have been directly used as the input of network-based transductive methods (e.g. *RANKS*), while the input for supervised feature-based inductive methods (e.g. *SVM*) has been constituted by the rows of the same adjacency matrices. In other words for each gene its input features are represented by the interactions and functional similarities with all the other genes of the network.

##### HPO DAG and annotations

Following the same experimental set-up of [10], we considered separately the three main sub-ontologies of the HPO (January 2014 release): *Organ Abnormality*, *Mode of Inheritance* and *Onset and Clinical Course*. *Organ Abnormality* is the main ontology and includes terms related to clinical abnormalities. *Mode of Inheritance* is a relatively small ontology and describes the inheritance pattern of the phenotypes. *Onset and Clinical Course* contains classes that describe typical modifiers of clinical symptoms, as the speed of progression, and the variability or the onset. For the sake of simplicity for the rest of the paper we refer to these sub-ontologies respectively as *Organ*, *Inheritance* and *Onset*. Following the experimental set-up of [10], we pruned the HPO terms having less than 10 annotations in the January 2014 release, thus resulting in DAGs having respectively 2134 (*Organ*), 13 (*Inheritance*) and 23 (*Onset*) terms.

#### Performance metrics

Since typically molecular biologists and physicians are interested in knowing both the set of genes associated with a certain HPO term and the phenotypic abnormalities associated with a particular human gene, we evaluated the results using two different performance metrics: (i) *term-centric* and (ii) *gene-centric*. These two types of evaluations were chosen to address the following related questions: (i) what are the genes associated with a specific abnormal phenotype? and (ii) what are the abnormal phenotypes associated with a particular gene?

*1. Gene-centric metric.* Precision (*Pr*), Recall (*Rc*) and the maximum achievable *F*−*s* *c* *o* *r* *e* (*F*_*max*_) using thresholds *τ*∈[0,1] are calculated as follows:

12 $$  Pr(\tau) = \frac{1}{N} \sum^{N}_{j=1} \frac{\sum_{i} \mathbbm{1}\left(i \in P_{g_{j}}(\tau) \wedge i \in T_{g_{j}}\right)}{\sum_{i} \mathbbm{1} \left(i \in P_{g_{j}}(\tau)\right)}  $$

13 $$  Rc(\tau) = \frac{1}{N} \sum^{N}_{j=1} \frac{\sum_{i} \mathbbm{1}\left(i \in P_{g_{j}}(\tau) \wedge i \in T_{g_{j}}\right)}{\sum_{i} \mathbbm{1}\left.\left(i \in T_{g_{j}}\right)\right)}  $$

14 $$  F_{max} = max_{\tau} \left \{ \frac{2 \cdot Pr(\tau) \cdot Rc(\tau)}{Pr(\tau) + Rc(\tau)} \right \}  $$

where *i* denotes an abnormal phenotype (HPO term) in the human ontology (excluding the root node), $P_{g_{j}} (\tau)$ denotes the set of terms that have a predicted scores greater than or equal to *τ* for a given gene *g*_*j*_, $T_{g_{j}}$ denotes the set of experimentally determined terms for a given gene *g*_*j*_, *N* the number of examples having at least one annotation with an HPO term and $\mathbbm {1}$ indicates a standard indicator function. In other words the *F*_*max*_ measure is the maximum hierarchical F-score achievable by “a posteriori” setting the optimal decision threshold [42].

We warn the reader that the hierarchical *F*−*s* *c* *o* *r* *e* as defined in eq. 14 provides an over-optimistic assessment of the hierarchical score. Indeed the threshold *τ* in eq. 14 is by definition “a posteriori” selected, by choosing the optimal *τ* ^∗^ that optimizes the *F*−*s* *c* *o* *r* *e* having a set of pre-computed scores. An unbiased evaluation should embed the selection of the optimal *τ* within the learning process. Nevertheless, we use this measure since it is the reference gene-centric metric within the computational biology community, as witnessed by its usage in the CAFA2 international challenge [42], and in the ongoing CAFA3 challenge.

*2. Term-centric metric.* For each term *i* we computed the classical Area Under the Receiver Operating Characteristic Curve (AUROC). The ROC curve is created by plotting the *sensitivity* (or *recall*) against *false positive rate* (or 1−*specificity*), measured at different threshold levels. The sensitivity (*Sn*) and specificity (*Sp*) at at a given score threshold *τ* for a particular functional term *i* are defined as:

15 $$  Sn_{i}(\tau) = \frac{\sum_{j} \mathbbm{1}\left(y_{i}^{g_{j}} > \tau \wedge g_{j} \in i\right)}{\sum_{j} \mathbbm{1}\left(g_{j} \in i\right)}  $$

16 $$  Sp_{i}(\tau) = \frac{\sum_{j} \mathbbm{1}\left(y_{i}^{g_{j}} \leq \tau \wedge g_{j} \notin i\right)}{\sum_{j} \mathbbm{1}\left(g_{j} \notin i\right)}  $$

where $y_{i}^{g_{j}} \in [0,1]$ is the predicted score (probability) that gene *g*_*j*_ is associated with the HPO term *i* and *τ*∈[0,1] is a given threshold, and $\mathbbm {1}$ indicates the standard indicator function. The ROC curve is computed by computing the sensitivity and 1 - specificity at different values of *τ*∈[0,1]. The AUROC can assume values in [0…1]; values close to 0.5 denote random predictions and values substantially larger than 0.5 denote good predictive ability.

For both measures, by averaging across genes or terms, we can obtain an overall picture of the prediction performance of the methods.

#### Experimental results

The best results have been obtained with the STRING network (“Data and experimental set-up” subsection) and among the different variants of the *TPR* algorithm, *TPR-W* (Eq. 8), with the *Threshold Free (TF)* strategy (Eq. 7) to select the set of “positive” children, achieved the best results. For this reason we firstly report the results obtained with STRING and the *TPR-W* ensemble, while the detailed results obtained with the other variants of the *TPR* algorithm as well as those obtained with the *UA* integrated network are available in the Additional files 4 and 5.

Table 1 summarizes the results achieved by the proposed hierarchical ensemble methods *HTD* and *TPR*, using as base learner *RANKS* (*TPR-W-RANKS* and *HTD-RANKS*) and a linear SVM (*TPR-W-SVM* and *HTD-SVM*). The results were compared with those achieved by state-of-the-art methods and the two flat methods used as base learner by the hierarchical ensembles (*RANKS* and *SVM*).

**Table 1:** Cross-validated prediction of genes associated with HPO terms of the *Organ, Inheritance* and Onset sub-ontology

	AUROC	*F*_*max*_	Precision	Recall
Organ sub-ontology				
*TPR-W-RANKS*	**0.89**	0.40	0.34	0.48
*TPR-W-SVM*	0.77	**0.44**	0.38	0.51
*HTD-RANKS*	0.88	0.37	0.30	0.49
*HTD-SVM*	0.75	0.43	0.37	0.49
*PHENOstruct*	0.73	0.42	0.35	**0.56**
*Clus-HMC-Ens*	0.65	0.41	**0.39**	0.43
*PhenoPPIOrth*	0.52	0.20	0.27	0.15
*SSVM →Dis→HPO*	0.49	0.23	0.16	0.41
*RANKS*	0.87	0.30	0.23	0.43
*SVM*	0.74	0.42	0.36	0.50
Inheritance sub-ontology				
*TPR-W-RANKS*	**0.91**	0.57	0.45	0.80
*TPR-W-SVMs*	0.82	0.69	0.59	0.82
*HTD-RANKS*	0.90	0.57	0.44	0.81
*HTD-SVMs*	0.81	0.69	0.59	0.82
*PHENOstruct*	0.74	**0.74**	**0.68**	0.81
*Clus-HMC-Ens*	0.73	0.73	0.64	**0.84**
*PhenoPPIOrth*	0.55	0.12	0.16	0.10
*SSVM →Dis→HPO*	0.46	0.11	0.07	0.25
*RANKS*	0.90	0.56	0.43	0.81
*SVMs*	0.82	0.69	0.59	0.82
Onset sub-ontology				
*TPR-RANKS*	**0.86**	0.44	0.33	**0.70**
*TPR-SVMs*	0.75	**0.48**	**0.38**	0.66
*HTD-RANKS*	**0.86**	0.42	0.30	0.69
*HTD-SVMs*	0.74	0.46	0.37	0.67
*PHENOstruct*	0.64	0.39	0.31	0.52
*Clus-HMC-Ens*	0.58	0.35	0.27	0.48
*PhenoPPIOrth*	0.53	0.25	0.25	0.24
*SSVM →Dis→HPO*	0.49	0.07	0.06	0.10
*RANKS*	0.83	0.41	0.30	0.67
*SVMs*	0.74	0.47	0.37	0.63

Average AUROC across terms and average *F*_*max*_, Precision and Recall across genes of *HTD, TPR-W* ensembles and state-of-the-art methods. Best results for each metric are highlighted in bold

*HTD* and *TPR* ensembles achieve statistically significant better results than state-of-the-art methods in terms of term-centric measures, independently of the base learner used: indeed, by applying the Wilcoxon rank sum test, the Bonferroni corrected p-value for multiple hypothesis testing resulted in a family wise error rate FWER ≤10^−4^.

Considering the hierarchical multi-label score (*F*_*max*_), *TPR-W-SVM* achieves significantly better results with respect to the other methods, with the only exception of the smallest HPO sub-ontology (*Inheritance*) that includes only 13 HPO terms (Table 1). Interestingly enough, the hierarchical ensemble methods are always able to improve the results of the flat methods used as base learner; in particular we have large improvement of the *F*_*max*_ when *RANKS* is used as base learner, while the improvement is smaller with the AUROC, for which *RANKS* alone achieves relatively high values. These results also show that the performance of hierarchical ensembles largely depends on that of the flat base learners: for instance *HTD-RANKS* and *TPR-W-RANKS* achieve a significantly larger average AUROC than *HTD-SVM* and *TPR-W-SVM* (Table 1). This is not surprising since the improvement introduced by hierarchical methods depends also on the ability of the underlying flat base learners to provide correct and at least partially consistent predictions. Indeed not always flat scores can be improved by hierarchical ensemble methods, as shown for instance in (Additional file 1: Figure S1): when very noisy or incorrect flat scores are provided, it is unlikely that hierarchical ensemble methods can improve the predictions. In the opposite case too, i.e. when flat scores are very close to optimal Bayes predictions, it is of course hard to improve performances for any method, including also *HTD-DAG* and *TPR-DAG*.

Overall, these results show that the proposed hierarchical ensemble methods are competitive with state-of-the-art methods such as *PHENOstruct* and *Clus-HMC-Ens* and moreover show that they can improve the results of different flat methods, such as the network-based semi-supervised *RANKS* algorithm and the supervised *SVM* classifier. Detailed results obtained with different variants of *TPR*, including *TPR-W*, *TPR-TF*, *TPR-T* and *TPR-D* (“Hierarchical true path rule (*TPR-DAG*) ensembles” subsection) are shown in additional file 4: *TPR-W* achieves most times the best results thanks to the tuning of the *w* parameter. The *w* parameter was selected through a classical double cross-validation procedure: the generalization performance was evaluated by the external cross-validation, while the “optimal” *w* parameter was chosen by internal cross-validation at each step of the external cross-validation. In this way we never accessed the examples of the test set for the selection of the *w* parameter. It is worth noting that the performance of other competing methods such as *PHENOstruct* or *Clus-HMC-Ens* could be enhanced by finely tuning their learning parameters. Nevertheless, *TPR-W* can further enhance its performance, by fine tuning the learning parameters of its base learners. We also observe that other *TPR* variants achieve competitive results without the need of tuning any parameter. For instance *TPR-D-SVM* shows an average *F*_*max*_ very close to that of *TPR-W-SVM* with the largest sub-ontology (*Organ*), and *TPR-TF-SVM* an average *F*_*max*_ very close to that of *TPR-W-SVM* with the *Inheritance* sub-ontology. Additional file 5 shows the results attained through the *UA* integrated network. Results are in most cases worse than those obtained with STRING data alone: this is not so surprising since STRING just combines different sources of information to construct the integrated network.

### HPO Prediction of newly annotated genes

In this section we assess the capacity of our proposed hierarchical ensemble methods to predict novel HPO annotations for human genes. To this end we used annotations of an old HPO release (January 2014) to predict the newly annotated genes of a recent HPO release (April 2016).

**Data sources** As data source we used the STRING 9.1 network, i.e one of the data sets used in the previous experiments (paragraph “Data sources”). Indeed the previous experiments as well as the experiments performed by [10] revealed that STRING 9.1 was the most informative source of information for the prediction of HPO terms. We did not use the most recent release of the STRING database (v.10, [49]), since we might introduce an indirect bias in the prediction, considering that STRING 10 was not available when the January 2014 HPO version was released.

**HPO DAG and annotations** The experiments presented here are based on the January 2014 HPO release (10,320 terms and 13,549 between-term relationships) to predict the newly annotated genes of the April 2016 HPO release (11,673 terms and 15,459 between-term relationships). Since in different releases some terms could have been removed, others changed or become obsolete, we mapped the old HPO terms to the new ones by parsing the annotation file of the January 2014 HPO release using as key the alt-ID taken from the obo file of the April 2016 HPO release. From the same HPO releases we downloaded all the corresponding gene-term associations. Then we pruned HPO terms having less than 10 annotations obtaining a final HPO DAG composed of 2445 terms and 3059 between-terms relationships, to avoid the prediction of HPO terms having a too few annotations for a reliable assessment.

#### Experimental set-up and performance metrics

We compared the generalization performance of *HTD* and *TPR* hierarchical ensemble methods versus PHENOstruct, the best performing state-of-the-art method in the previous set of experiments (“Prediction of Human Phenotype Ontology terms” subsection).

We denote with *T* the set of genes having at least 1 annotation with an HPO term of the “old” January 2014 HPO release (2804 genes) and with *S* the set of newly annotated genes, i.e. genes having at least one new annotation in the “new” April 2016 HPO release, but previously unannotated in the January 2014 HPO release (608 genes). Hence we have that *S*∩*T*=*∅*. We used the set *T* as training set and the set *S* as test set, and we applied a classical hold-out procedure to assess the capability of predicting newly annotated genes using only the annotations of the previous HPO release. In this way we predicted the newly annotated 608 genes of the test set, having on the average about 100 annotations per gene, distributed across 2445 HPO terms. For the *HTD* and *TPR* methods we used the SVMs as base learners. To evaluate the performance of PHENOstruct, we downloaded and adapted the freely available C++ PHENOstruct code to perform the hold-out procedure described above.

As performance metrics we used the same *gene-centric* and *term-centric* measures mentioned in “Performance metrics” subsection. In addition we added a further *term-centric* measure: the Area Under the Precision Recall Curve (AUPRC), to take into account the imbalance of annotated vs. unannotated HPO terms [50]. Unlike the previous experimental part (“Prediction of Human Phenotype Ontology terms” subsection), in the experiments presented here we considered the whole HPO, without splitting it up in its main sub-ontologies.

#### Experimental results

**Fig. 6:**
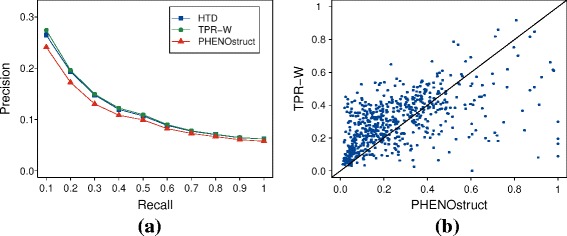
(**a**) Compared precision at different recall levels averaged across 2444 HPO terms. (**b**) Scatter plot of *F*_*max*_ values. Each point represent one of the 608 genes of the test set. *PHENOstruct* values are in abscissa, *TPR-W* values in ordinate

Table 2 shows that *TPR-W* and *HTD* are able to predict newly annotated genes, even if with a certain decay in the overall performance, as expected, with respect to the cross-validated results of Table 1. Hierarchical ensemble methods attain significantly better results than *PHENOstruct* both in terms of average AUPRC and *F*_*max*_ (Wilcoxon paired rank sum test, *p*-value <10^−9^), while *PHENOstruct* achieves the best AUROC results. It is worth noting that the precision of *TPR-W* and *HTD* is higher than that of *PHENOstruct* at any recall level (Fig. 6a), and these results are confirmed also by the “per-gene” hierarchical *F*_*max*_ score: *TPR-W* “wins” with 431 and “loses” with 177 human genes (Fig. 6b). Results obtained with other different *TPR* variants are comparable with those obtained by *TPR-W* (see Additional file 6). We observe that on this task the SVM performance is close to that of the hierarchical ensemble methods. If on the one hand *TPR-W* achieves better results than the SVM, on the other hand not always the difference is statistically significant: more precisely the difference is statistically significant with the *F*_*max*_ measure, while for the per-term metric *AUPRC* no statistical difference is detected. These results show that on this task is difficult to improve also on relatively simple baseline methods, and more research is needed to significantly enhance the performance.

**Table 2:** Prediction of newly annotated human genes

Method	AUROC	AUPRC	*F*_*max*_	Precision	Recall
*SVM*	0.6506	0.1230	0.3774	0.3457	0.4155
*HTD*	0.6464	0.1207	0.3794	**0.3581**	0.4033
*TPR-W*	0.6512	0.1237	**0.3826**	0.3512	0.4202
*PHENOstruct*	**0.6661**	0.1089	0.3635	0.3040	**0.4519**

Average AUROC and AUPRC across terms and average *F*_*max*_, Precision and Recall across genes. Results significantly better than the others according to the Wilcoxon Rank Sum test (*α*=10^−9^) are highlighted in bold

**Fig. 7:**
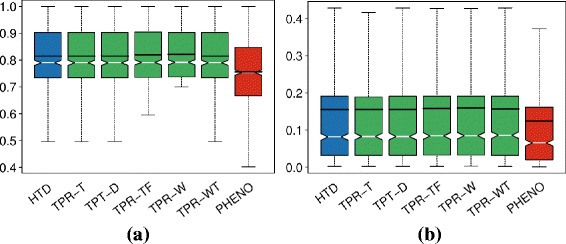
Distribution of the AUROC and AUPRC values across the best predicted terms (778 HPO terms). **a** AUROC, **b** AUPRC. *HTD* and different *TPR* variants are compared with *PHENOstruct*

We note that the best method (*TPR-W*) for about half of the newly annotated genes (296) obtained a reasonable accuracy, i.e. *F*_*max*_>0.3, as well as a relatively large area under the curve (AUROC >0.7) for about 800 HPO terms. Results limited to these best predicted genes and terms are summarized in Table 3). Figure 7 shows the distribution of the best “per-term” AUROC and AUPRC results of *HTD* and different variants of *TPR*, and in the Additional file 7 are shown their best results in terms of average AUROC, AUPRC and *F*_*max*_.

**Table 3:** Prediction of newly annotated human genes considering only the best predictions

Method	AUROC	AUPRC	*F*_*max*_	Precision	Recall
*SVM*	0.8208	0.1589	0.4727	0.4560	0.4908
*HTD*	0.8155	0.1551	0.4716	0.4429	0.5042
*TPR-W*	**0.8219**	0.1594	**0.4793**	**0.4572**	0.5037
*PHENOstruct*	0.7565	0.1241	0.4297	0.3583	**0.5366**

Results significantly better than the others according to the Wilcoxon Rank Sum test (*α*=10^−9^) are highlighted in bold

**Fig. 8:**
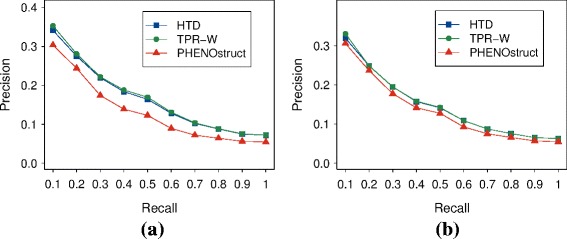
Precision at different recall levels of the newly annotated genes, considering only the best predicted terms. **a** Results considering only the HPO terms predicted with AUROC >0.7 by *TPR-W* (778 terms); **b** results considering only the HPO terms predicted with AUROC >0.7 by *PHENOstruct* (852 terms)

While the results of Fig. 7 and those shown in Additional file 7 are biased in favor of TPR-W (only the genes and terms best predicted by *TPR-W* are included), Fig. 8 shows that hierarchical ensemble methods achieve competitive results in terms of precision at any recall level independently if the best predicted HPO terms are selected with respect to *TPR-W* or *PHENOstruct* best predictions.

The empirical computational time of hierarchical ensemble methods is significantly lower than that of state-of-the-art joint kernel structured output methods. Indeed the overall training and test time for the hold-out processing is about 12 min with *HTD* and about 18 h with *PHENOstruct* using an Intel Xeon CPU E5-2630 2.60GHz with 128 GB of RAM. The overall computation time with *TPR-W* is significantly larger than *HTD* (about 3 h), due to the tuning of the *w* parameter of the algorithm, but in any case significantly lower than *PHENOstruct*. It is worth noting that the overall computational time of the hierarchical ensemble methods depend on the training time of the base learner, and may of course vary with the complexity of the base learner used. We can also observe that *TPR-W* usually achieves slightly better results than *HTD* (Tables 1 and 3), but if the computational time is an issue, we can safely use *HTD* or other *TPR* variants with lower computational complexity, such as *TPR-TF* or *TPR-D*, with only a small decay in performance.

### New “candidate genes” predicted by the hierarchical ensemble

In this section we provide a list of currently unannotated genes that could be possible candidate for novel annotations. To this purpose we at first selected a list of the HPO terms best predicted by the hierarchical ensemble methods, and then we selected among these HPO terms, those currently unannotated genes that achieved a hierarchical score larger than those of the annotated genes. More precisely, by exploiting the scores computed by *TPR-W* in the hold-out experiments (“HPO Prediction of newly annotated genes” subsection), we selected genes not annotated in the April 2016 release of HPO, but predicted to be annotated by our method to specific HPO terms, thus resulting to be possible candidate genes for being annotated with that term.

First of all, by considering the hierarchical ensemble *TPR-W-SVM*, that achieved the best results in the detection of newly annotated genes (“HPO Prediction of newly annotated genes” subsection), we selected the HPO terms best predicted by our method, i.e. terms predicted with AUROC larger than a given threshold. To this end we considered the 130 HPO terms that obtained an AUROC value higher than 0.95 (Additional file 8). From this set of HPO terms we selected the most specific nodes of the hierarchy, that is those that correspond to the leaves of the HPO DAG (65 HPO terms).

Finally we selected for these most specific and best predicted terms, the genes candidate to be annotated for that term. To this end, for each of the selected 65 HPO terms we adopted the following procedure:

1. Sort in descending order all the genes on the basis of the *TPR-W-SVM* scores;
2. Select the first top 5 ranked genes (set *S*);
3. Select the top ranked genes in *S* annotated for the HPO term (set *A*⊆*S*);
4. Select the maximum score $\bar {s}$ among the annotated genes belonging to *A*;
5. The candidate genes are those unannotated genes in *S* (the top ranked 5 genes) having a score larger or equal than $\bar {s}$, or all the genes in *S* if *A*=*∅*.

As a result, for each HPO term we selected the top ranked unannotated genes having a *TPR-W-SVM* score higher or equal than the highest score achieved by the genes annotated for that term. In other words the procedure selects those unannotated genes that *TPR-W* strongly thinks to be annotated for that term. The procedure limits the analysis to the top 5 ranked genes for each best predicted and most specific term, in order to provide a list of genes well-characterized about their abnormal phenotype and possibly reliable, according to the performance of the *TPR-W-SVM* ensemble. It is worth noting that following the above procedure for selecting the candidate genes, not necessarily for each HPO leaf term we have always five candidate genes. Indeed it may happen that for a given HPO term an annotated gene fills, e.g., the third position in the rank, and hence for that term we will have only two candidate genes. The full list of the the genes candidate to be annotated for the best predicted and most specific 65 HPO terms are available in the Additional file 9.

We note that some of the newly predicted associations gene-HPO term have been confirmed in the most recent HPO release (March 2017). For instance the predicted association of the gene *XRCC2* with HPO term *HP:0100760* (Clubbing of toes) has been confirmed in the March 2017 HPO release, as well as the association of the gene *LIPE* (that encodes the protein LIPE E) with the HPO term *HP:0000831* (Insulin-resistant diabetes mellitus). Interestingly enough *LIPE* is correlated also with the disease LIPE-related familial partial Lipodystrophy (*ORPHA:435660*). The March 2017 HPO release also confirmed the predicted association of gene *IGF2*, that encodes a member of the insulin family of polypeptide growth factor, with the phenotype *HP:0100631* (Neoplasm of the adrenal gland), and moreover recent works postulated the association of *IGF2* with adrenal tumors ed in particular with adrenocortical carcinomas and pheochromocytomas [51]. Besides the aforementioned evidence of associations, the novel predicted gene-HPO term pairs show a clear relationship with specific diseases, according to the most recent literature. For example the human gene *ECHS1*, that encodes the enzyme that catalyzes the second step of the mitochondrial fatty acid *β*-oxidation (FAO) pathway, is correlated with the phenotype *HP:0004359* (Abnormality of fatty-acid metabolism) and from literature is also known being associated with FAO disorders and in particular with the Leigh syndrome [52]. Moreover the predicted association between the Complement factor B (*CFB*) and the term *HP:0002725* (Systemic lupus erythematosus) is supported by recent literature, since *CFB* is an important activator of the alternative complement pathway and increasing evidence supports reducing factor B as a potential novel therapy to lupus nephritis [53]. Another strong evidence of associations between the gene–HPO term pair predicted by our ensemble method and the corresponding disease is given by the work of Hersh et al. [54]. In this work the authors demonstrated the importance of *TGFBR3* gene (encoding the transforming growth factor (TGF)-beta type III receptor) in the Chronic Obstructive Pulmonary Disease (COPD), confirming the prediction made by our method which associated *TGFBR3* gene with the HPO term Emphysema (*HP:0002097*). Furthermore the predicted association between the gene *BARD1* and Nephroblastoma, also known as Wilms’ tumor (*HP:0002667*), is supported in the very recent work of Fu et al. [55], in which the authors claim that the *BARD1* gene polymorphism is significantly associated with increased nephroblastoma risk. As stated in [56], frameshift mutations in the *MSH3* gene play an important role in the development of breast cancer (*HP:0003002*), supporting in this way the selfsame gene–HPO term association predicted by our methods. The predicted association between the *CAD* gene (encoding for a tri-functional enzyme involved in the pyrimidine biosynthesis) and the phenotype *HP:0004353* (Abnormality of pyrimidine metabolism) is confirmed by the fact that a mutation in *CAD* impairs *de novo* pyrimidine biosynthesis [57]. Another interesting example is represented by the predicted association between the *COX10* gene (Cytochrome c Oxidase) and the phenotype Abnormal mitochondria in muscle tissue (*HP:0008316*), supported by literature evidence that correlates the *COX10* dysfunction with mitochondrial disease [58]. Overall, the novel annotations in the recent (March 2017) HPO release as well as recent bio-medical literature support the novel predictions obtained with our hierarchical ensemble methods.

## Conclusions

The experimental results show that hierarchical ensemble methods are able to predict associations between genes and abnormal phenotypes with results competitive with state-of-the-art algorithms. The low computational complexity of the hierarchical correction step of both *HTD* and *TPR* (linear with respect the number of nodes of the HPO) enables its efficient application using different types of base learners. Indeed we showed that the proposed hierarchical algorithms are able to improve the predictions of both semi-supervised flat methods, such as the *RANKS* algorithm, that resulted one of the top ranked method in the recent CAFA2 challenge for HPO term prediction [42], and of supervised methods such as SVM. By exploiting the modular structure of *HTD* and *TPR*, we speculate that in principle any flat method, used as base learner within our proposed hierarchical algorithms, can in principle improve its performance for the prediction of HPO terms. We also proved that both *HTD* and *TPR* always provide consistent predictions that obey the true path rule, a fundamental fact to assure biologically coherent predictions of HPO terms. Moreover we provided a list of currently unannotated genes that could be possible candidate for novel HPO annotations, and we showed that several predicted gene - HPO term associations have been confirmed in the current HPO release (March 2017) and in recent bio-medial literature. Since for several disorders no disease genes have been discovered (e.g. for about half of Mendelian diseases no causative genes are known [59]), our methods can contribute to the discovery of such genes, and to unravel the full spectrum of phenotypes associated with them.

Even if the overall results show the effectiveness of state-of-the-art methods for the prediction of HPO terms, the estimated absolute value of both *AUPRC* and *F*_*max*_ for genome-wide HPO term predictions cannot be considered fully satisfactory, showing that there is room for further research on this topic.

Finally we point out that our methods are general enough to be applied to other prediction problems in computational biology characterized by taxonomies structured as directed acyclic graphs. For instance, the proposed approach could be safely applied to the prediction of Gene Ontology terms, assuring biological consistency of the predictions and at the same time likely improving predictions obtained with flat learning methods. Furthermore, since a tree is a DAG, our proposed algorithms can be also safely applied to tree-structured taxonomies, such as the FunCat [8].

## Additional files

Additional file 1 Correctness of the predictions: real data example. (PDF 651 kb)

Additional file 2 Consistency of the predictions: intuitive example. (PDF 85 kb)

Additional file 3 Consistency of the predictions: details and theorem proofs. (PDF 153 kb)

Additional file 4 Prediction of Human Phenotype Ontology terms: detailed experimental results using STRING network. (PDF 92.3 kb)

Additional file 5 Prediction of Human Phenotype Ontology terms: detailed experimental results using UA integrated network. (PDF 92.3 kb)

Additional file 6 HPO Prediction of Newly Annotated Genes: detailed experimental results. (PDF 77.4 kb)

Additional file 7 HPO Prediction of Newly Annotated Genes: detailed experimental results considering only the best predictions for the newly annotated genes. (PDF 77.6 kb)

Additional file 8 Best predicted HPO terms sorted in descending order on the basis of AUROC. (PDF 80.3 kb)

Additional file 9 Top 5 candidate genes for each of the 65 leaf nodes showed in Additional file 8. (PDF 141 kb)

## Abbreviations

AUPRC: Area under the precision recall curve
AUROC: Area under the receiver operating characteristic curve
CAFA: Critical assessment of protein function annotation DAG: Direct-acyclic-graph
HPO: Human phenotype ontology
HTD: Hierarchical top-down
RANKS: RAnking of nodes with kernelized score functions
SVM: Support vector machines
TPR: True path rule
UA: Unweighted average network integration
