# Supplementary material for: Prediction of Human Phenotype Ontology terms by means of hierarchical ensemble methods
Source: BMC Bioinformatics. 2017 Oct 12;18:449. doi: 10.1186/s12859-017-1854-y (PMC5639780; doi:10.1186/s12859-017-1854-y)
Supplement: Supplementary file 1 — Correctness of the predictions: real data example. (PDF 651 kb) [file 12859_2017_1854_MOESM1_ESM.pdf]

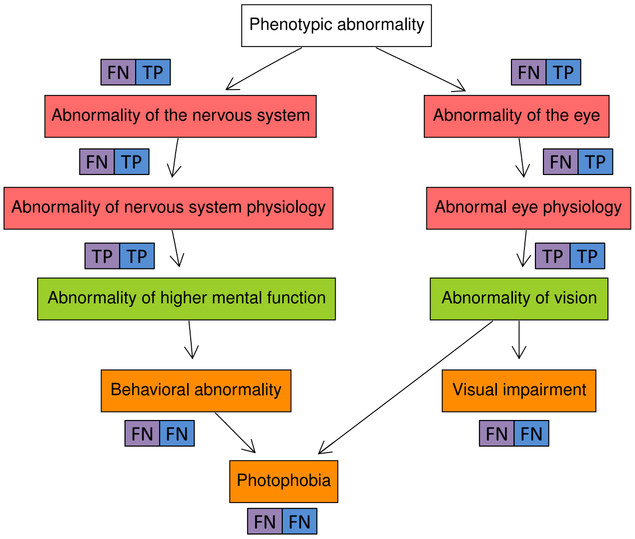

(a)

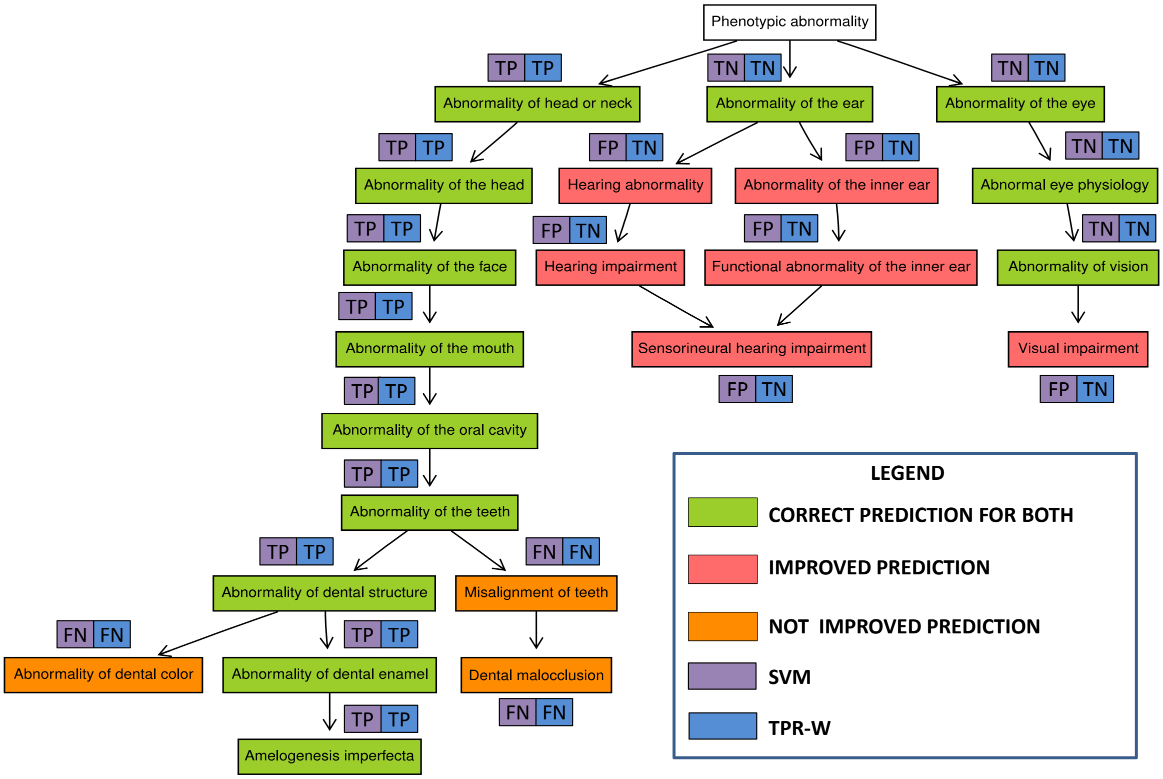

(b)

Additional Figure 1: Flat and hierarchical (*TPR-W*) HPO predictions for the gene *RGS9* (a) and for the gene *ENAM* (b). The boxes close to each HPO term display the correct (TP or TN) or incorrect (FP or FN) predictions made respective by flat-SVM (purple rectangles) and by hierarchical-TPR-W (blue rectangles). Green rectangles represent correct predictions for both flat and hierarchical methods; light-red rectangles represent the predictions that the hierarchical method was able to correct respect to flat method ($FP \to TN$ or $FN \to TP$), while the orange rectangles represent the incorrect predictions that the hierarchical method was not able to correct respect to the flat method. We used the January 2014 HPO release as ground truth for the gene-phenotype associations.
