# Supplementary material for: Prediction of Human Phenotype Ontology terms by means of hierarchical ensemble methods
Source: BMC Bioinformatics. 2017 Oct 12;18:449. doi: 10.1186/s12859-017-1854-y (PMC5639780; doi:10.1186/s12859-017-1854-y)
Supplement: Supplementary file 2 — Consistency of the predictions: intuitive example. (PDF 85 kb) [file 12859_2017_1854_MOESM2_ESM.pdf]

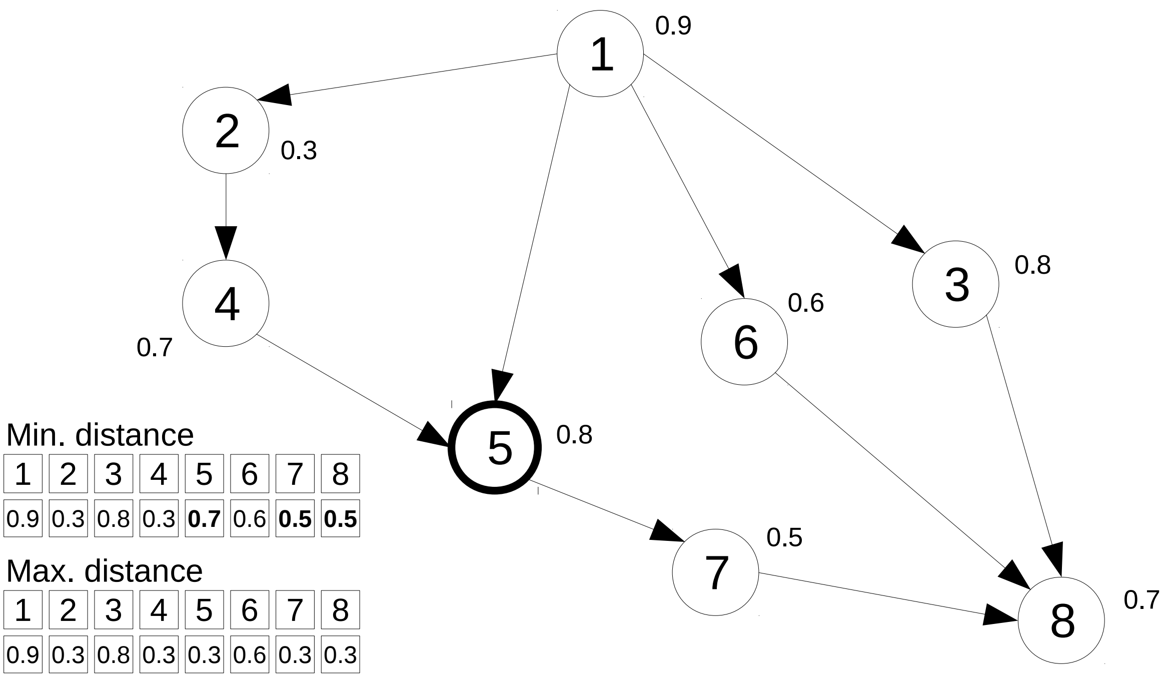

Additional Figure 2: Levels defined in terms of the minimum distance from the root (node 1) lead to inconsistent predictions. The small numbers close to nodes correspond to the $\hat{y}_i$ scores of the flat predictions. The Hierarchical top-down scores obtained respectively by crossing the levels according to the minimum and the maximum distance from the root are shown in the bottom-left. Scores in boldface represent inconsistent predictions.
