# Supplementary material for: Prediction of Human Phenotype Ontology terms by means of hierarchical ensemble methods
Source: BMC Bioinformatics. 2017 Oct 12;18:449. doi: 10.1186/s12859-017-1854-y (PMC5639780; doi:10.1186/s12859-017-1854-y)
Supplement: Supplementary file 3 — Consistency of the predictions: details and theorem proofs. (PDF 153 kb) [file 12859_2017_1854_MOESM3_ESM.pdf]

Consistency of the predictions: details and theorem proofs

Additional file 2 provides a simple example to show that we need a per level traversal of the DAG, in which levels are defined in the sense of the maximum distance from the root, to obtain consistent predictions that obey the "true path rule". Indeed looking at the *HTD-DAG* scores obtained respectively with the minimum and maximum distance from the root (bottom-left of Additional Figure 2 of the additional file 2), we see that only the maximum distance preserves the consistency of the predictions. For instance, focusing on node 5, by traversing the DAG levels according to the minimum distance from the root, we have that the level of node 5 is 1 ($\psi^{min}(5) = 1$) and in this case by applying the *HTD* rule (eq. 2 of the main manuscript) the flat score $\hat{y}_5 = 0.8$ is wrongly modified with the *HTD* ensemble score $\bar{y}_5 = 0.7$. If we instead traverse the DAG levels according to the maximum distance from the root, we have $\psi^{max}(5) = 3$ and the *HTD* ensemble score is correctly set to $\bar{y}_5 = 0.3$. In other words at the end of the *HTD*, by traversing the levels according to the minimum distance we have $\bar{y}_5 = 0.7 > \bar{y}_4 = 0.3$. Therefore a child node has a score larger the score of its parent and the true path rule is not preserved. On the contrary by traversing the levels according to the maximum distance we achieve $\bar{y}_5 = 0.3 \leq \bar{y}_4 = 0.3$ and the true path rule consistency is assured. This is due to the fact that by adopting the minimum distance when we visit node 5, node 4 has not just been visited, and hence the value 0.4 has not been transmitted by node 2 to node 4; on the contrary if we visit the DAG according to the maximum distance all the ancestors of node 5 (including node 4) have just been visited and the score 0.4 is correctly transmitted to node 5 along the path $2 \rightarrow 4 \rightarrow 5$.

To prove the consistency of the predictions of the *HTD-DAG* algorithm, we first introduce a property of the level function ψ. We recall here the definition of ψ, just given in the main paper. If $p(r, i)$ represents a path from the root node r and a node $i \in V$, $l\,(p(r, i))$ the length of $p(r, i)$, $\mathcal{L} = \{0, 1, \ldots, \xi\}$ the set of observed levels, with ξ the maximum node level, then $\psi : V \longrightarrow \mathcal{L}$ is a level function which assigns each node $i \in V$ to its level $\psi(i)$:

$$\psi(i) = \max_{p(r,i)} \; l\,(p(r, i)) \tag{1}$$

Nodes $\{i|\psi(i) = 0\}$ correspond to the root nodes, $\{i|\psi(i) = 1\}$ is the set of nodes with a maximum path length from the root (distance) equal to 1, and $\{i|\psi(i) = \xi\}$ are nodes that lie at a maximum distance ξ from the root.

To prove the consistency, we need the following lemma:

Lemma 1 *Given a DAG $G =< V, E >$, a level function ψ that assigns to each node its maximum path length from the root, it holds that $\forall i \in V$, $\psi(j) < \psi(i)$ $\forall j \in par(i)$.*

Proof The proof is based on the optimal-substructure property holding for the longest path problem in DAGs, that is a longest path between two vertices contains other longest path within it [1]. Indeed, let $\bar{p}(r, i)$ be the longest path from $r = root(G)$ to node $i \in V$, and suppose that there exists $j \in par(i)$ such that $\psi(j) \geq \psi(i)$. Let $\bar{p}(r, j)$ be the path between r and j whose length is $\psi(j)$ (that is the longest path between them). Note that the path $p(r, j)$ does not contain the node i, otherwise the DAG would contain a cycle. By adding the edge (j, i) to $\bar{p}(r, j)$, we obtain a path from r to i whose length is $\psi(j) + 1 > \psi(i)$, which contradicts the hypothesis that $\bar{p}(r, i)$ is the longest path between nodes r and i. □

By using Lemma 1, we can prove that the the top-down visit of the DAG obeys the true path rule:

Theorem 1 *Given a DAG $G =< V, E >$, a level function ψ that assigns to each node its maximum path length from the root and the set of* HTD-DAG *flat predictions $\hat{\boldsymbol{y}} =< \hat{y}_1, \hat{y}_2, \dots, \hat{y}_{|V|} >$, the top-down hierarchical correction of the* HTD-DAG *algorithm assures that the set of ensemble predictions $\bar{\boldsymbol{y}} =< \bar{y}_1, \bar{y}_2, \dots, \bar{y}_{|V|} >$ satisfies the following property:*

$$\forall i \in V,\ j \in par(i) \Rightarrow \bar{y}_j \geq \bar{y}_i$$

Proof For an arbitrary node $i \in V$, when it is processed by the top-down step of *HTD-DAG* algorithm, we may have two basic cases:

1 $i \in root(G)$. By applying the HTD rule (Section 2.3 of the main manuscript):

$$\bar{y}_i := \begin{cases} \hat{y}_i & \text{if} \quad i \in root(G) \\ \min_{j \in par(i)} \bar{y}_j & \text{if} \quad \min_{j \in par(i)} \bar{y}_j < \hat{y}_i \\ \hat{y}_i & \text{otherwise} \end{cases} \tag{2}$$

we set $\bar{y}_i := \hat{y}_i$ and the property $j \in par(i) \Rightarrow \bar{y}_j \geq \bar{y}_i$ trivially holds, since $par(i) = \emptyset$.

2 $i \notin root(G)$. We may have two cases:

(a) $\hat{y}_i \leq \min_{j \in par(i)} \bar{y}_j$. In this case the rule (2) sets $\bar{y}_i := \hat{y}_i$ and hence it holds that $j \in par(i) \Rightarrow \bar{y}_j \geq \bar{y}_i$.

(b) $\hat{y}_i > \min_{j \in par(i)} \bar{y}_j$. In this case by applying (2) of the main manuscript we have $\bar{y}_i := min_{j \in par(i)} \bar{y}_j$ and hence also in this case the property $j \in par(i) \Rightarrow \bar{y}_j \geq \bar{y}_i$ holds.

Summarizing, in all cases we have that $j \in par(i) \Rightarrow \bar{y}_j \geq \bar{y}_i$, after the node i has been processed. Moreover, we note that for the currently processed node i both $\bar{y}_i$ and $\bar{y}_j$, $j \in par(i)$ will not be further changed by the "per level" top-down visit of the *HTD-DAG* algorithm. Indeed, the score $\bar{y}_i$ is modified only once, since each node is visited exactly one time (each node belongs to one and only one level of the hierarchy); moreover, since the visit is top-down, Theorem 1 implies that parent nodes are processed before their children, and hence also the scores $\bar{y}_j$ of the nodes $j \in par(i)$ will not be further changed, since $j \in par(i)$ have just been visited and their scores $\bar{y}_j$ have just been set before visiting node i. As a consequence, once a node i is visited the property $j \in par(i) \Rightarrow \bar{y}_j \geq \bar{y}_i$ will hold till to the end of the algorithm.

Finally, since the top-down step of the algorithm visits each node exactly one time, at the end of this step the property $j \in par(i) \Rightarrow \bar{y}_j \geq \bar{y}_i$ holds for each node $i \in V$. □

From Theorem 1 it is easy to prove that the consistency of the predictions holds for all the ancestors of a given node $i \in V$.

Corollary 1 *Given a DAG $G =< V, E >$, the level function ψ and the set of flat predictions $\hat{\boldsymbol{y}} =< \hat{y}_1, \hat{y}_2, \dots, \hat{y}_{|V|} >$, the* HTD-DAG *algorithm assures that for the set of ensemble predictions $\bar{\boldsymbol{y}} =< \bar{y}_1, \bar{y}_2, \dots, \bar{y}_{|V|} >$ the following property holds: $\forall i \in V,\ j \in anc(i) \Rightarrow \bar{y}_j \geq \bar{y}_i$.*

Proof The corollary can be proven by "reductio ad absurdum" from Theorem 1. We suppose that for an arbitrary node i does exist a node $z \in anc(i)$ such that $\bar{y}_z < \bar{y}_i$. Let us consider all the edges (k, l) included in the path $\bar{p}(z, i)$ connecting node z with node i. Without loss of generality, we focus on a specific path, since we can repeat the same reasoning for any path connecting z with i. We claim that

$\exists(k,l) \in \bar{p}(z,i)$ such that $\bar{y}_k < \bar{y}_l$, and we show this again by "reductio ad absurdum". By absurd we suppose that $\forall(k,l) \in \bar{p}(z,i)$ we have $\bar{y}_k \geq \bar{y}_l$. By transitivity along the path $\bar{p}(z,i)$, we obtain that $\bar{y}_z \geq \bar{y}_i$, but this contradicts our first hypothesis that $\bar{y}_z < \bar{y}_i$ and hence it does exist an edge $(k,l) \in \bar{p}(z,i)$ such that $\bar{y}_k < \bar{y}_l$. But for Theorem 1 it is not possible that $\bar{y}_k < \bar{y}_l$, since $k \in par(l)$. Since this contradiction comes from the assumption that does exist a node $z \in anc(i)$ such that $\bar{y}_z < \bar{y}_i$, it follows that $\forall i \in V,\ j \in anc(i) \Rightarrow \bar{y}_j \geq \bar{y}_i$. □

Independently of the choice of the positive children, the following consistency theorem holds for *TPR-DAG*:

Theorem 2 *Given a DAG $G =< V, E >$, a set of flat predictions $\hat{\boldsymbol{y}} =< \hat{y}_1, \hat{y}_2, \ldots, \hat{y}_{|V|} >$ for each class associated to each node $i \in \{1, \ldots, |V|\}$, the* TPR-DAG *algorithm assures that for the set of ensemble predictions $\bar{\boldsymbol{y}} =< \bar{y}_1, \bar{y}_2, \ldots, \bar{y}_{|V|} >$ the following property holds: $\forall i \in V,\ j \in anc(i) \Rightarrow \bar{y}_j \geq \bar{y}_i$.*

The proof is substantially the same of Theorem 1 and is omitted for brevity.

It is worth noting that the following properties hold for *HTD-DAG* and *TPR-DAG* algorithms:

Lemma 2 *Given a DAG $G =< V, E >$, a set of flat predictions $\hat{\boldsymbol{y}} =< \hat{y}_1, \hat{y}_2, \ldots, \hat{y}_{|V|} >$ for each class associated to each node $i \in \{1, \ldots, |V|\}$, a set of ensemble predictions $\bar{\boldsymbol{y}} =< \bar{y}_1, \bar{y}_2, \ldots, \bar{y}_{|V|} >$ for the* HTD-DAG *and a set of ensemble predictions $\tilde{\boldsymbol{y}} =< \tilde{y}_1, \tilde{y}_2, \ldots, \tilde{y}_{|V|} >$ for the* TPR-DAG *with "positive" children selected according to eq. 7 of the main manuscript, we have that $\forall i \in V, \quad \tilde{y}_i \geq \bar{y}_i$.*

The proof is based on the fact that the bottom-up step of *TPR-DAG* can only increment the scores $\tilde{y}_i$ with respect to the flat predictions $\hat{y}_i$. Hence the successive top-down step of the *TPR-DAG* starts from higher scores than that of the *HTD-DAG*, and the applied top-down procedure is the same for both algorithms.

A good property of *TPR-DAG* is that its sensitivity is always equal or better than that of the *HTD-DAG*:

Theorem 3 *The* TPR-DAG *ensemble algorithm with "positive" children selected according to eq. 7 of the main manuscript achieves always a sensitivity equal or higher than the* HTD-DAG *ensemble algorithm.*

Proof From Lemma 2 we have that $\forall i \in V, \quad \tilde{y}_i \geq \bar{y}_i$. Hence the *TPR-DAG* ensemble algorithm, with respect to the *HTD-DAG* algorithm:

a) increments or maintains equal the number of true positives;
b) decreases or maintains equal the number of false negatives.

By definition of the sensitivity *TPR-DAG* achieves a sensitivity equal or higher than the *HTD-DAG*. □

Unfortunately there is no guarantee that the precision of *TPR-DAG* is always larger or equal than that of the *HTD-DAG* algorithm.

References

1. Dasgupta, S., Papadimitriou, C., Vazirani, U.: Algorithms. McGraw Hill, Boston (2008)
