# Supplementary material for: Prediction of Human Phenotype Ontology terms by means of hierarchical ensemble methods
Source: BMC Bioinformatics. 2017 Oct 12;18:449. doi: 10.1186/s12859-017-1854-y (PMC5639780; doi:10.1186/s12859-017-1854-y)
Supplement: Supplementary file 4 — Prediction of Human Phenotype Ontology terms: detailed experimental results using STRING network. (PDF 92.3 kb) [file 12859_2017_1854_MOESM4_ESM.pdf]

Additional Table 1. Average AUROC across terms and average F_{max}, Precision and Recall across genes of HTD and TPR ensemble variants using RANKS and SVMs as base learner and the STRING network. Results of "flat" RANKS and SVMs are also reported. Results are estimated through 5-fold cross-validation. For each sub-ontology and each metric best results are highlighted in bold. Results significantly better than all the others methods according to the Wilcoxon Rank Sum test ($\alpha = 10^{-6}$) are underlined.

Subontology	Method	AUROC	F_{max}	Precision	Recall
**Organ**	*RANKS*	0.8540	0.3048	0.2349	0.4338
	SVM	0.7440	0.4188	0.3598	0.5008
	HTD-RANKS	0.8812	0.3743	0.3041	0.4865
	HTD-SVM	0.7475	0.4249	0.3739	0.4919
	TPR-T-RANKS	0.8604	0.3775	0.3048	0.4973
	TPR-T-SVM	0.7602	0.4318	0.3850	0.4931
	TPR-D-RANKS	0.8598	0.3986	0.3409	0.4798
	TPR-D-SVM	0.7723	0.4341	**0.3883**	0.4933
	TPR-TF-RANKS	0.8609	0.3752	0.3056	0.4858
	TPR-TF-SVM	0.7584	0.4330	0.3696	<u>**0.5227**</u>
	TPR-W-RANKS	<u>**0.8857**</u>	0.3999	0.3429	0.4805
	TPR-W-SVM	0.7713	**0.4354**	0.3782	0.5107
**Inheritance**	*RANKS*	0.8983	0.5601	0.4292	0.8061
	SVM	0.8164	0.6835	0.5876	0.8167
	HTD-RANKS	0.9005	0.5682	0.4392	0.8045
	HTD-SVM	0.8101	0.6869	0.5892	0.8234
	TPR-T-RANKS	0.9043	0.5718	0.4469	0.7950
	TPR-T-SVM	0.8187	0.6894	0.5939	0.8219
	TPR-D-RANKS	0.9044	0.5718	0.4469	0.7950
	TPR-D-SVM	0.8187	0.6894	0.5939	0.8219
	TPR-TF-RANKS	**0.9150**	0.5385	0.4024	0.8139
	TPR-TF-SVM	0.8254	0.6848	0.5854	**0.8248**
	TPR-W-RANKS	0.9147	0.5718	0.4470	0.7950
	TPR-W-SVM	0.8187	**0.6898**	**0.5951**	0.8208
**Onset**	*RANKS*	0.8325	0.4143	0.3025	0.6568
	SVM	0.7365	0.4656	0.3689	0.6309
	HTD-RANKS	**0.8605**	0.4174	0.2999	0.6861
	HTD-SVM	0.7433	0.4584	0.3651	0.6156
	TPR-T-RANKS	0.8575	0.4184	0.2926	<u>**0.7432**</u>
	TPR-T-SVM	0.7432	0.4616	0.3691	0.6171
	TPR-D-RANKS	0.8563	0.4372	0.3214	0.6868
	TPR-D-SVM	0.7434	0.4618	0.3594	0.6528
	TPR-TF-RANKS	0.8580	0.4218	0.3046	0.6853
	TPR-TF-SVM	0.7463	0.4668	0.3670	0.6410
	TPR-W-RANKS	0.8573	0.4401	0.3264	0.7004
	TPR-W-SVM	0.7442	<u>**0.4770**</u>	**0.3741**	0.6644
