# Supplementary material for: Prediction of Human Phenotype Ontology terms by means of hierarchical ensemble methods
Source: BMC Bioinformatics. 2017 Oct 12;18:449. doi: 10.1186/s12859-017-1854-y (PMC5639780; doi:10.1186/s12859-017-1854-y)
Supplement: Supplementary file 5 — Prediction of Human Phenotype Ontology terms: detailed experimental results using UA integrated network. (PDF 92.3 kb) [file 12859_2017_1854_MOESM5_ESM.pdf]

Additional Table 2. Average AUROC across terms and average F_{max}, Precision and Recall across genes of HTD and TPR ensemble variants using RANKS and SVMs as base learner and the UA integrated network. Results are estimated through 5-fold cross-validation. For each sub-ontology and each metric best results are highlighted in bold. Results significantly better than all the others methods according to the Wilcoxon Rank Sum test ($\alpha = 10^{-6}$) are underlined.

Subontology	Method	AUROC	F_{max}	Precision	Recall
**Organ**	*RANKS*	0.8493	0.3106	0.2407	0.4377
	SVM	0.7128	0.1668	0.1688	0.1648
	HTD-RANKS	0.8446	0.3411	0.2717	0.4583
	HTD-SVM	0.8328	0.3155	0.2370	0.4718
	TPR-T-RANKS	**<u>0.8646</u>**	0.3575	0.2933	0.4586
	TPR-T-SVM	0.8240	0.3155	0.2370	0.4718
	TPR-D-RANKS	0.8408	**<u>0.3773</u>**	**<u>0.3231</u>**	0.4538
	TPR-D-SVM	0.7883	0.3209	0.2800	0.3782
	TPR-TF-RANKS	0.8420	0.3547	0.2880	0.4615
	TPR-TF-SVM	0.7060	0.2359	0.2074	0.2736
	TPR-W-RANKS	0.8377	0.3620	0.3025	0.4515
	TPR-W-SVM	0.8238	0.3171	0.2343	**<u>0.4916</u>**
**Inheritance**	*RANKS*	0.8715	0.5986	0.4709	0.8215
	SVM	0.7637	0.1668	0.1688	0.1648
	HTD-RANKS	**0.8846**	0.6034	0.4709	0.8396
	HTD-SVM	0.7991	0.5429	0.3885	0.9008
	TPR-T-RANKS	0.8708	0.6066	**0.4795**	0.8276
	TPR-T-SVM	0.7909	0.5444	0.3892	0.9063
	TPR-D-RANKS	0.8659	0.6064	0.4793	0.8278
	TPR-D-SVM	0.7919	0.5441	0.3889	0.9063
	TPR-TF-RANKS	0.8675	0.6029	0.4757	0.8228
	TPR-TF-SVM	0.7546	0.4480	0.2972	**0.9099**
	TPR-W-RANKS	0.8636	**0.6070**	0.4769	0.8355
	TPR-W-SVM	0.7900	0.5448	0.3897	0.9059
**Onset**	*RANKS*	0.8159	0.4494	0.3821	0.5455
	SVM	0.7025	0.3978	0.2717	0.7421
	HTD-RANKS	**0.8253**	0.4466	0.3563	0.5981
	HTD-SVM	0.7781	0.4492	0.3374	0.6719
	TPR-T-RANKS	0.8249	0.4631	0.3791	0.6021
	TPR-T-SVM	0.7511	0.4517	0.3385	0.6813
	TPR-D-RANKS	0.8170	**0.4684**	0.3839	0.6077
	TPR-D-SVM	0.7513	0.4519	0.3394	0.6780
	TPR-TF-RANKS	0.8148	0.4553	0.3782	0.5718
	TPR-TF-SVM	0.6788	0.3983	0.2714	**<u>0.7483</u>**
	TPR-W-RANKS	0.7901	0.4682	**<u>0.3914</u>**	0.5856
	TPR-W-SVM	0.7496	0.4522	0.3404	0.6757
