# Supplementary material for: Prediction of Human Phenotype Ontology terms by means of hierarchical ensemble methods
Source: BMC Bioinformatics. 2017 Oct 12;18:449. doi: 10.1186/s12859-017-1854-y (PMC5639780; doi:10.1186/s12859-017-1854-y)
Supplement: Supplementary file 6 — HPO Prediction of Newly Annotated Genes: detailed experimental results. (PDF 77.4 kb) [file 12859_2017_1854_MOESM6_ESM.pdf]

Additional Table 3. Prediction of newly annotated genes. Average AUROC and AUPRC across 2444 HPO terms and average F_{max}, Precision and Recall of HTD and TPR ensemble variants across the newly annotated 608 genes. Best results for each metric are highlighted in bold.

Methods \ Meas.	AUROC	AUPRC	F_{max}	Precision	Recall
HTD	0.6464	0.1207	0.3794	0.3581	0.4033
TPR-T	0.6466	0.1209	0.3795	0.3580	0.4036
TPR-D	0.6465	0.1211	0.3794	**0.3584**	0.4030
TPR-TF	0.6498	0.1224	0.3812	0.3560	0.4101
TPR-W	**0.6512**	**0.1237**	**0.3826**	0.3512	**0.4202**
