# Supplementary material for: Prediction of Human Phenotype Ontology terms by means of hierarchical ensemble methods
Source: BMC Bioinformatics. 2017 Oct 12;18:449. doi: 10.1186/s12859-017-1854-y (PMC5639780; doi:10.1186/s12859-017-1854-y)
Supplement: Supplementary file 7 — HPO Prediction of Newly Annotated Genes: detailed experimental results considering only the best predictions for the newly annotated genes. (PDF 77.6 kb) [file 12859_2017_1854_MOESM7_ESM.pdf]

**Additional Table 4.** Comparison among HTD and TPR ensemble variants considering only the best predictions for the newly annotated genes. Average AUROC and AUPRC across terms and average $F_{max}$, Precision and Recall across genes considering only HPO terms with AUROC > 0.7 (778 terms) and $F_{max} > 0.3$ (296 genes). Best results for each metric are highlighted in bold.

| Meas. / Methods | AUROC | AUPRC | $F_{max}$ | Precision | Recall |
|---|---|---|---|---|---|
| *HTD* | 0.8155 | 0.1551 | 0.4716 | 0.4429 | 0.5042 |
| *TPR-T* | 0.8155 | 0.1553 | 0.4714 | 0.4443 | 0.5021 |
| *TPR-D* | 0.8155 | 0.1554 | 0.4714 | 0.4436 | 0.5030 |
| *TPR-TF* | 0.8200 | 0.1577 | 0.4765 | 0.4419 | **0.5171** |
| *TPR-W* | **0.8219** | **0.1594** | **0.4793** | **0.4572** | 0.5037 |
