# Supplementary material for: Prediction of Human Phenotype Ontology terms by means of hierarchical ensemble methods
Source: BMC Bioinformatics. 2017 Oct 12;18:449. doi: 10.1186/s12859-017-1854-y (PMC5639780; doi:10.1186/s12859-017-1854-y)
Supplement: Supplementary file 8 — Best predicted HPO terms sorted in descending order on the basis of AUROC. (PDF 80.3 kb) [file 12859_2017_1854_MOESM8_ESM.pdf]

Additional Table 5: The best predicted HPO terms sorted in descending order on the basis of AUROC. Depth stands for the maximum distance of a given term from the root node (in the considered HPO graph the longest path from a node to a root is 14). Distance from leaves indicates the minimum distance of a given node from one of the leaves of the HPO-DAG. A value equal to 0 is assigned to the leaves, 1 to nodes with distance 1 from a leaf and so on.

HPO ID	HPO term	AUROC	Depth	Distance from Leaves
HP:0004942	Aortic aneurysm	1.000	7	0
HP:0100760	Clubbing of toes	1.000	9	0
HP:0000114	Proximal tubulopathy	0.9984	9	0
HP:0003557	Increased variability in muscle fiber diameter	0.9984	6	0
HP:0002040	Esophageal varix	0.9967	7	0
HP:0002913	Myoglobinuria	0.9967	6	0
HP:0003642	Type I transferrin isoform profile	0.9967	8	0
HP:0006477	Abnormality of the alveolar ridges	0.9967	7	1
HP:0006846	Acute encephalopathy	0.9967	5	1
HP:0006965	Acute necrotizing encephalopathy	0.9967	6	0
HP:0008316	Abnormal mitochondria in muscle tissue	0.9967	6	0
HP:0004339	Abnormality of sulfur amino acid metabolism	0.9967	5	0
HP:0004944	Cerebral aneurysm	0.9967	8	0
HP:0002725	Systemic lupus erythematosus	0.9967	5	0
HP:0001659	Aortic regurgitation	0.9951	7	0
HP:0004353	Abnormality of pyrimidine metabolism	0.9951	4	0
HP:0000831	Insulin-resistant diabetes mellitus	0.9934	6	0
HP:0001019	Erythroderma	0.9934	7	0
HP:0002223	Absent eyebrow	0.9934	9	0
HP:0003160	Abnormal isoelectric focusing of serum transferrin	0.9934	7	1
HP:0004481	Progressive macrocephaly	0.9934	9	0
HP:0010459	True hermaphroditism	0.9934	7	0
HP:0012345	Abnormal glycosylation	0.9934	4	4
HP:0012346	Abnormal protein glycosylation	0.9934	5	3
HP:0012347	Abnormal protein N-linked glycosylation	0.9934	6	2
HP:0003521	Disproportionate short-trunk short stature	0.9926	6	0
HP:0010996	Abnormality of monocarboxylic acid metabolism	0.9918	4	0
HP:0000991	Xanthomatosis	0.9901	6	0
HP:0003645	Prolonged partial thromboplastin time	0.9901	4	0
HP:0009161	Aplasia/Hypoplasia of the middle phalanx of the 5th finger	0.9885	11	0
HP:0010932	Abnormality of nucleobase metabolism	0.9885	3	1
HP:0003076	Glycosuria	0.9869	7	0
HP:0011016	Abnormality of urine glucose concentration	0.9869	6	1
HP:0002304	Akinesia	0.9868	6	0
HP:0003953	Absent forearm bone	0.9868	9	1
HP:0003974	Absent radius	0.9868	10	0
HP:0009822	Aplasia involving forearm bones	0.9868	9	1
HP:0003215	Dicarboxylic aciduria	0.9868	8	0
HP:0010995	Abnormality of dicarboxylic acid metabolism	0.9868	4	1
HP:0004219	Abnormality of the middle phalanx of the 5th finger	0.9852	10	1
HP:0002085	Occipital encephalocele	0.9835	8	0
HP:0000677	Oligodontia	0.9819	10	0
HP:0001218	Autoamputation	0.9819	3	0
HP:0003254	Abnormality of DNA repair	0.9819	4	0
HP:0100864	Short femoral neck	0.9819	10	0
HP:0009027	Foot dorsiflexor weakness	0.9810	7	0
HP:0009108	Foot dorsiflexor weakness	0.9802	9	1

HPO ID	HPO term	AUROC	Depth	Distance from Leaves
HP:0002905	Hyperphosphatemia	0.9802	5	0
HP:0001436	Abnormality of the foot musculature	0.9794	5	1
HP:0002839	Urinary bladder sphincter dysfunction	0.9786	7	0
HP:0009617	Abnormality of the distal phalanx of the thumb	0.9786	11	0
HP:0002097	Emphysema	0.9785	5	0
HP:0100631	Neoplasm of the adrenal gland	0.9781	5	0
HP:0000073	Ureteral duplication	0.9769	6	0
HP:0002181	Cerebral edema	0.9769	8	0
HP:0002557	Hypoplastic nipples	0.9753	5	0
HP:0004359	Abnormality of fatty-acid metabolism	0.9753	4	0
HP:0009720	Adenoma sebaceum	0.9736	8	0
HP:0010615	Angiofibromas	0.9736	7	1
HP:0003233	Hypoalphalipoproteinemia	0.9730	7	0
HP:0010980	Hyperlipoproteinemia	0.9727	6	0
HP:0003002	Breast carcinoma	0.9723	5	0
HP:0001480	Freckling	0.9720	6	0
HP:0005293	Venous insufficiency	0.9720	5	0
HP:0005613	Aplasia/hypoplasia of the femur	0.9720	8	2
HP:0006443	Patellar aplasia	0.9719	9	0
HP:0002025	Anal stenosis	0.9711	8	0
HP:0002298	Absent hair	0.9711	6	1
HP:0007431	Congenital ichthyosiform erythroderma	0.9703	8	1
HP:0001992	Organic aciduria	0.9703	7	1
HP:0006498	Aplasia/Hypoplasia of the patella	0.9691	8	1
HP:0003310	Abnormality of the odontoid process	0.9686	6	1
HP:0009888	Abnormality of secondary sexual hair	0.9678	5	0
HP:0003311	Hypoplasia of the odontoid process	0.9671	7	0
HP:0010979	Abnormality of the level of lipoprotein cholesterol	0.9661	5	1
HP:0002898	Embryonal neoplasm	0.9655	4	1
HP:0004311	Abnormality of macrophages	0.9653	6	0
HP:0001974	Leukocytosis	0.9638	6	0
HP:0100578	Lipoatrophy	0.9638	5	0
HP:0000625	Cleft eyelid	0.9631	8	0
HP:0007361	Abnormality of the pons	0.9625	8	0
HP:0010981	Hypolipoproteinemia	0.9625	6	1
HP:0002667	Nephroblastoma (Wilms tumor)	0.9615	8	0
HP:0002612	Congenital hepatic fibrosis	0.9576	6	0
HP:0012144	Abnormality of cells of the monocyte/macrophage lineage	0.9570	4	1
HP:0011794	Embryonal renal neoplasm	0.9569	7	1
HP:0009733	Glioma	0.9555	7	1
HP:0002967	Cubitus valgus	0.9541	7	0
HP:0012072	Aciduria	0.9539	6	2
HP:0010286	Abnormality of the salivary glands	0.9539	7	0
HP:0010675	Abnormal foot bone ossification	0.9539	7	0
HP:0010899	Abnormality of aspartate family amino acid metabolism	0.9539	5	0
HP:0002922	Increased CSF protein	0.9522	6	0
HP:0000855	Insulin resistance	0.9516	5	1
