# Supplementary material for: Prediction of Human Phenotype Ontology terms by means of hierarchical ensemble methods
Source: BMC Bioinformatics. 2017 Oct 12;18:449. doi: 10.1186/s12859-017-1854-y (PMC5639780; doi:10.1186/s12859-017-1854-y)
Supplement: Supplementary file 9 — Top 5 candidate genes for each of the 65 leaf nodes showed in Additional file 8. (PDF 141 kb) [file 12859_2017_1854_MOESM9_ESM.pdf]

Additional Table 6: Top 5 candidate genes for each of the 65 leaf nodes showed in the additional file 8. See the main manuscript for more details about the selection of the candidate genes for each HPO leaf term.

HPO ID	HPO term	Entrez Gene ID	Gene Symbol
HP:0004942	Aortic aneurysm	72	ACTG2
HP:0100760	Clubbing of toes	7516	XRCC2
HP:0000114	Proximal tubulopathy	1892	ECHS1
HP:0003557	Increased variability in muscle fiber diameter	8516	ITGA8
		3694	ITGB6
HP:0002040	Esophageal varix	85440	DOCK7
		7049	TGFBR3
HP:0002913	Myoglobinuria	1962	EHHADH
		1892	ECHS1
HP:0003642	Type I transferrin isoform profile	201595	STT3B
		1109	AKR1C4
		199857	ALG14
HP:0006965	Acute necrotizing encephalopathy	4697	NDUFA4
		4539	MT-ND4L
HP:0008316	Abnormal mitochondria in muscle tissue	4697	NDUFA4
		51204	TACO1
		1352	COX10
		4539	MT-ND4L
		4538	MT-ND4
HP:0004339	Abnormality of sulfur amino acid metabolism	1757	SARDH
		55811	ADCY10
		1137	CHRNA4
		271	AMPD2
		272	AMPD3
HP:0004944	Cerebral aneurysm	1757	SARDH
		55811	ADCY10
		91137	SLC25A46
		271	AMPD2
		272	AMPD3
HP:0002725	Systemic lupus erythematosus	629	CFB
HP:0001659	Aortic regurgitation	27229	TUBGCP4
		2239	GPC4
		3340	NDST1
		57822	GRHL3
		5378	PMS1
HP:0004353	Abnormality of pyrimidine metabolism	790	CAD
		7516	XRCC2
		580	BARD1
		10747	MASP2
		58484	NLRC4
HP:0000831	Insulin-resistant diabetes mellitus	3991	LIPE
		3764	KCNJ8
		5260	PHKG1
HP:0001019	Erythroderma	3149	HMGB3
		7516	XRCC2
		5657	PRTN3
		28952	CCDC22
		2224	FDPS

HPO ID	HPO term	Entrez Gene ID	Gene Symbol
HP:0002223	Absent eyebrow	9463	PICK1
		389549	FEZF1
HP:0004481	Progressive macrocephaly	4697	NDUFA4
		4538	MT-ND4
		54539	NDUFB11
		4539	MT-ND4L
HP:0010459	True hermaphroditism	54585	LZTFL1
		9786	KIAA0586
		11020	IFT27
		57822	GRHL3
		26281	FGF20
HP:0003521	Disproportionate short-trunk short stature	54567	DLL4
		3371	TNC
		9096	TBX18
HP:0010996	Abnormality of monocarboxylic acid metabolism	790	CAD
HP:0000991	Xanthomatosis	9971	NR1H4
		4047	LSS
		3991	LIPE
		80347	COASY
		4744	NEFH
HP:0003645	Prolonged partial thromboplastin time	2266	FGG
		3026	HABP2
		10747	MASP2
		733	C8G
		199857	ALG14
HP:0009161	Aplasia/Hypoplasia of the middle phalanx of the 5th finger	26585	GREM1
		2535	FZD2
		57216	VANGL2
HP:0003076	Glycosuria	140628	GATA5
		388753	COA6
HP:0002304	Akinesia	1139	CHRNA7
		9037	SEMA5A
		54567	DLL4
		8506	CNTNAP1
		3763	KCNJ6
HP:0003974	Absent radius	6909	TBX2
		26585	GREM1
		7468	WHSC1
		23129	PLXND1
		6997	TDGF1
HP:0003215	Dicarboxylic aciduria	1962	EHHADH
		1892	ECHS1
		3417	IDH1
		4329	ALDH6A1
		126129	CPT1C
HP:0002085	Occipital encephalocele	284217	LAMA1
		1109	AKR1C4
		85301	COL27A1
		8516	ITGA8
		3694	ITGB6
HP:0000677	Oligodontia	25885	POLR1A
		2535	FZD2
		7161	TP73
		7049	TGFBR3

HPO ID	HPO term	Entrez Gene ID	Gene Symbol
		28952	CCDC22
HP:0001218	Autoamputation	4916	NTRK3
		51164	DCTN4
		10715	CERS1
		127833	SYT2
		10087	COL4A3BP
HP:0003254	Abnormality of DNA repair	7516	XRCC2
		4437	MSH3
		7469	NELFA
		580	BARD1
		79991	STN1
HP:0100864	Short femoral neck	11020	IFT27
		2239	GPC4
		283375	SLC39A5
		2817	GPC1
		22978	NT5C2
HP:0009027	Foot dorsiflexor weakness	56776	FMN2
		4744	NEFH
		5859	QARS
HP:0002905	Hyperphosphatemia	844	CASQ1
		26281	FGF20
		6809	STX3
		80055	PGAP1
HP:0002839	Urinary bladder sphincter dysfunction	25894	PLEKHG4
		81570	CLPB
		6327	SCN2B
		23025	UNC13A
		27445	PCLO
HP:0009617	Abnormality of the distal phalanx of the thumb	2619	GAS1
		26585	GREM1
		57216	VANGL2
		22978	NT5C2
		84976	DISP1
HP:0002097	Emphysema	5450	POU2AF1
		3105	HLA-A
		7049	TGFBR3
HP:0100631	Neoplasm of the adrenal gland	1021	CDK6
		7468	WHSC1
		3481	IGF2
HP:0000073	Ureteral duplication	9091	PIGQ
		7161	TP73
		126129	CPT1C
		1962	EHHADH
		284098	PIGW
HP:0002181	Cerebral edema	790	CAD
		4697	NDUFA4
		4538	MT-ND4
		4539	MT-ND4L
		1892	ECHS1
HP:0002557	Hypoplastic nipples	9091	PIGQ
		6909	TBX2
		4040	LRP6
		284098	PIGW
		120	ADD3

HPO ID	HPO term	Entrez Gene ID	Gene Symbol
HP:0004359	Abnormality of fatty-acid metabolism	1962	EHHADH
		1892	ECHS1
		126129	CPT1C
		4329	CPT1C
		57468	SLC12A5
HP:0009720	Adenoma sebaceum	2475	MTOR
		4437	MSH3
		2272	FHIT
		5293	PIK3CD
		5378	PMS1
HP:0003233	Hypoalphalipoproteinemia	9971	NR1H4
		3991	LIPE
		5260	PHKG1
		27229	TUBGCP4
		5446	PON3
HP:0010980	Hyperlipoproteinemia	9971	NR1H4
		5446	PON3
		8694	DGAT1
		5445	PON2
HP:0003002	Breast carcinoma	4437	MSH3
HP:0001480	Freckling	4437	MSH3
		5378	PMS1
		7161	TP73
		1021	CDK6
		1031	CDKN2C
HP:0005293	Venous insufficiency	54567	DLL4
		1303	COL12A1
		1399	CRKL
		3371	TNC
		6678	SPARC
HP:0006443	Patellar aplasia	7994	KAT6A
HP:0002025	Anal stenosis	6909	TBX2
		80055	PGAP1
HP:0009888	Abnormality of secondary sexual hair	6909	TBX2
		7161	TP73
		4744	NEFH
		4047	LSS
		6862	T
HP:0003311	Hypoplasia of the odontoid process	3371	TNC
		4774	NFIA
		22926	ATF6
		8239	USP9X
		26137	ZBTB20
HP:0004311	Abnormality of macrophages	2214	FCGR3A
		330	BIRC3
		4671	NAIP
		58484	NLRC4
		6809	STX3
HP:0001974	Leukocytosis	3551	IKBKB
		3932	LCK
		5657	PRTN3
		58484	NLRC4
		330	BIRC3

HPO ID	HPO term	Entrez Gene ID	Gene Symbol
HP:0100578	Lipoatrophy	3991	LIPE
		23022	PALLD
		844	CASQ1
		29119	CTNNA3
		6006	RHCE
HP:0000625	Cleft eyelid	23129	PLXND1
		246243	RNASEH1
		9463	PICK1
		25885	POLR1A
		10512	SEMA3C
HP:0007361	Abnormality of the pons	5297	PI4KA
		9091	PIGQ
		284098	PIGW
		80055	PGAP1
		163786	SASS6
HP:0002667	Nephroblastoma (Wilms tumor)	580	BARD1
HP:0002612	Congenital hepatic fibrosis	11020	CYP4B1
		54585	LZTFL1
		85440	85440
		51164	DCTN4
		55690	PACS1
HP:0002967	Cubitus valgus	26585	GREM1
		26281	FGF20
HP:0010286	Abnormality of the salivary glands	4437	MSH3
		7161	TP73
		5378	PMS1
		2272	FHIT
		3417	IDH1
HP:0010675	Abnormal foot bone ossification	4047	LSS
		3149	HMGB3
		440275	EIF2AK4
		389549	FEZF1
		22926	ATF6
HP:0010899	Abnormality of aspartate family amino acid metabolism	1757	SARDH
		55811	ADCY10
		271	AMPD2
		272	AMPD3
		91137	SLC25A46
HP:0002922	Increased CSF protein	57468	SLC12A5
		3106	HLA-B
		3105	HLA-A
		4179	CD46
		283375	SLC39A5
